# Using data from online geocoding services for the assessment of environmental obesogenic factors: a feasibility study

**DOI:** 10.1186/s12942-019-0177-9

**Published:** 2019-06-07

**Authors:** Maximilian Präger, Christoph Kurz, Julian Böhm, Michael Laxy, Werner Maier

**Affiliations:** 1 Institute of Health Economics and Health Care Management, Helmholtz Zentrum München – German Research Center for Environmental Health (GmbH), Ingolstädter Landstraße 1, 85758 Neuherberg, Germany; 2 German Center for Diabetes Research, Neuherberg, Germany

**Keywords:** Obesogenic environment, Geocoding services, Validation, Diabetes

## Abstract

**Background:**

The increasing prevalence of obesity is a major public health problem in many countries. Built environment factors are known to be associated with obesity, which is an important risk factor for type 2 diabetes. Online geocoding services could be used to identify regions with a high concentration of obesogenic factors. The aim of our study was to examine the feasibility of integrating information from online geocoding services for the assessment of obesogenic environments.

**Methods:**

We identified environmental factors associated with obesity from the literature and translated these factors into variables from the online geocoding services Google Maps and OpenStreetMap (OSM). We tested whether spatial data points can be downloaded from these services and processed and visualized on maps. True- and false-positive values, false-negative values, sensitivities and positive predictive values of the processed data were determined using search engines and in-field inspections within four pilot areas in Bavaria, Germany.

**Results:**

Several environmental factors could be identified from the literature that were either positively or negatively correlated with weight outcomes in previous studies. The diversity of query variables was higher in OSM compared with Google Maps. In each pilot area, query results from Google showed a higher absolute number of true-positive hits and of false-positive hits, but a lower number of false-negative hits during the validation process. The positive predictive value of database hits was higher in OSM and ranged between 81 and 100% compared with a range of 63–89% for Google Maps. In contrast, sensitivities were higher in Google Maps (between 59 and 98%) than in OSM (between 20 and 64%).

**Conclusions:**

It was possible to operationalize obesogenic factors identified from the literature with data and variables available from geocoding services. The validity of Google Maps and OSM was reasonable. The assessment of environmental obesogenic factors via geocoding services could potentially be applied in diabetes surveillance.

**Electronic supplementary material:**

The online version of this article (10.1186/s12942-019-0177-9) contains supplementary material, which is available to authorized users.

## Background

Obesity, commonly defined as a body mass index (BMI) of ≥ 30 kg/m^2^ in adults [1], is the result of a complex multifactorial relationship (e.g. genetic, socioeconomic, and cultural factors) [2]. The prevalence of obesity is affected by lifestyle habits, consumption patterns as well as the urban development [2]. Since the 1980s, the prevalence of obesity has risen considerably and doubled in many countries [3]. Furthermore, a high BMI seems to be associated with a significant proportion of mortality and disability cases [4, 5]. Obesity is therefore recognized as a serious worldwide epidemic.

A number of severe health conditions are correlated with being very overweight, e.g. cardiovascular disease and hypertension, but in particular type 2 diabetes mellitus (T2DM) [6], which is the second leading cause of BMI-related deaths in 2015 [4]. Furthermore, obesity and overweight are the single most relevant predictors for T2DM [7]. Because some studies revealed the simultaneous spread of obesity and diabetes, the term ‘diabesity’ has been used in the literature in order to illustrate the close connectedness [8].

The built environment, comprising buildings, spaces and products generated or influenced by humans, has a strong influence on promoting or preventing diseases [9, 10]. The built environment can act on three different scales: the macro level describes the sprawl or the compactness of a region on a higher aggregated level, e.g. at the nationwide level, whereas the meso level is concerned with the community or neighbourhood environment, in which the access to certain facilities is of major interest. The micro level constitutes a person-related perspective, for example regarding qualities of urban design, and is often connected with the concept of walkability [11]. Factors of the built environment may contribute to obesity, for example via the availability of unhealthy food or the absence of green spaces [12], and consequently create obesogenic environments. Following Swinburn and colleagues [13], obesogenic environments can be described as ‘the sum of influences that the surroundings, opportunities, or conditions of life have on promoting obesity in individuals or populations’.

In order to evaluate features of the built environment, tools based on the use of geographic information systems (GIS) have been developed using remote sensing techniques applicable as desk-based approaches [14]. In the past, researchers have shown great interest in commercial data within GIS-based analyses [15, 16]. Recently, freely available data from online geocoding services such as Google Maps and OpenStreetMap (OSM) have become increasingly popular [17, 18]. These services are often accessed via embedded application programming interfaces (APIs) to search data within the geographical databases, e.g. for food-related data [19]. These freely available data can be further applied to assess the environmental risk of the development of obesity by describing high- and low-risk geographical areas originating from the accumulation of obesogenic and protective environmental factors [20]. Further applications of such data could refer to environmental pollution or geographical access to primary health care [21, 22].

The aim of our study was to examine the feasibility of integrating information from online geocoding services into the assessment of environmental obesogenic factors which could potentially be used for diabetes surveillance. Diabetes risk has often been estimated e.g. using data from national surveys, but also from administrative data [23]. Thus secondary data from online geocoding services could be a potential complementary data source for diabetes surveillance. Considering this, two steps were required: First, we checked whether obesogenic and protective factors can be derived from the literature and translated into variables from online geocoding services. Second, we compared Google Maps and OSM regarding their validity and reliability of queried data.

## Methods

### Design of the validation process

**Fig. 1:**
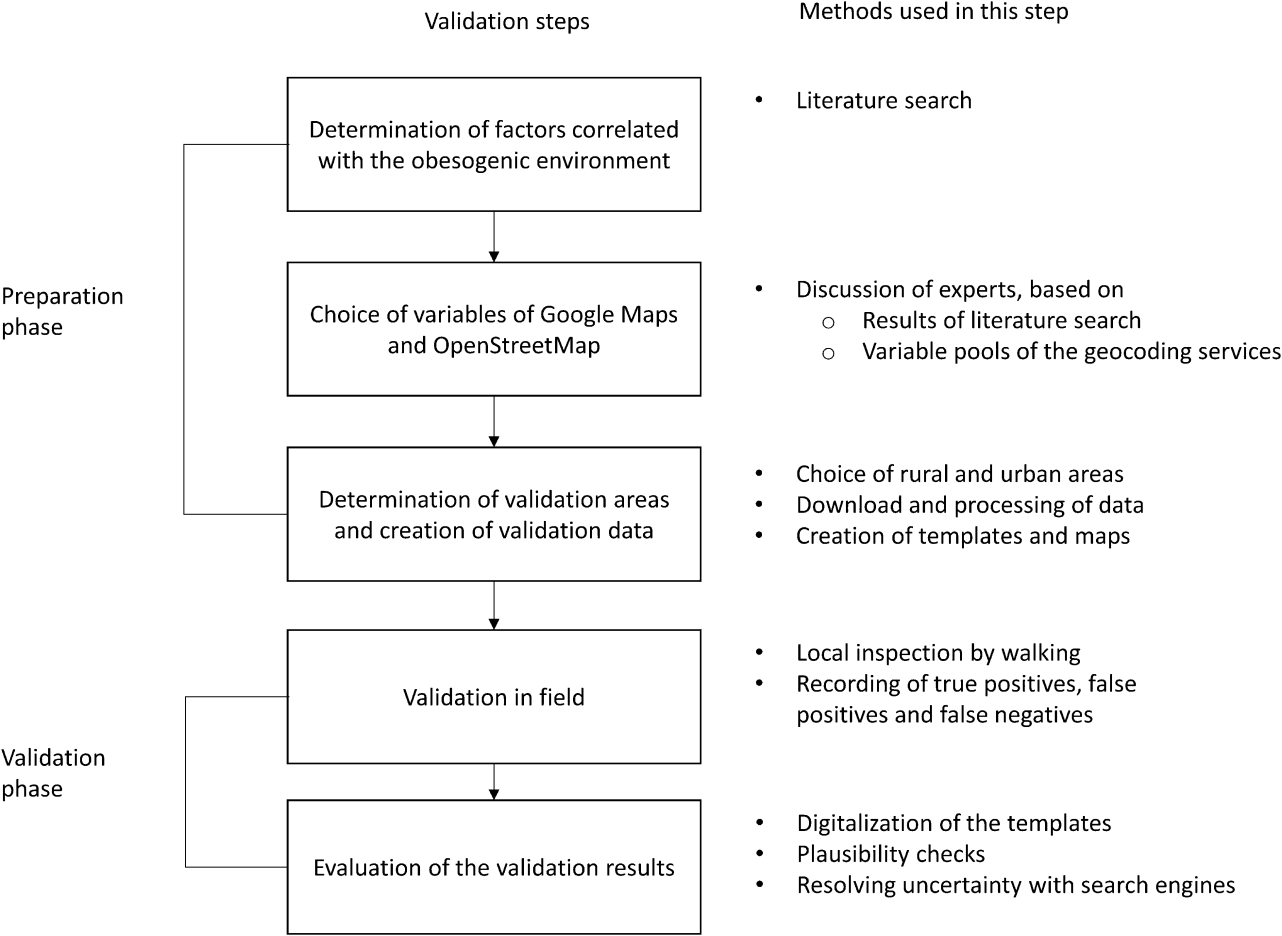
Flow chart of the validation process

To prepare subsequent validations, we initially identified environmental factors correlated with obesity from the literature. Based on these results and on expert discussions, we have chosen variables from Google Maps and OSM and downloaded these for four regions in Bavaria, Germany. Subsequently, these downloaded data points were validated in the field and by using search engines. An overview of the methods applied during the two phases of preparation and validation is shown in Fig. 1, and further details are provided below.

### Literature search and extraction of variables

We applied a search strategy within PubMed using the search terms ‘obesogenic’, ‘environmental factors’, ‘systematic’ and ‘review’. After screening the results, two reviews were determined to be relevant for our analysis. The first review by Mackenbach and colleagues [24] provided a systematic search strategy and identified correlates of environmental factors with obesity. The second publication was a review of GIS methods by Jia and colleagues [25], in which correlations of variables with weight status and obesity were described. Following Mackenbach et al. [24], we created a table in order to summarize the factors from our literature search. In a first step, we extracted environmental factors from the studies covered by the two reviews. In a second step, we grouped the publications describing these environmental factors and extracted and summarized information from these publications in order to determine their correlation with obesity.

Subsequently, we extended the systematic search strategy provided by Mackenbach et al. in order to identify recent additional studies within PubMed, EMBASE, Web of Science, Cochrane Library, PsychInfo and Google Scholar. We completed the variable table with the additionally identified publications, and information from these studies was used to update the correlations of the environmental factors.

### Definition of correlation

For each given environmental factor, we summed up the numbers of studies describing a positive and significant correlation with obesity. Analogously, we counted the numbers of studies describing negative and significant correlations with obesity for the same given factor. Subsequently, we defined this factor as overall positively correlated if at least three publications could be found and if the ratio of the number of positive correlations for the factor divided by the number of negative correlations for the same factor was 2 or higher. Dividing by 0 in this sense can be interpreted as causing infinity. Studies showing no significant correlation were not taken into account. Analogously, if the number of negative correlations divided by the number of positive correlations equals 2 or more, we assumed the factor to be overall negatively correlated. Otherwise, we supposed that no association existed. For example, if a factor was described with a positive correlation in five publications and with a negative correlation in 12 publications, an overall negative correlation was assumed as 12/5 ≥ 2. This calculation procedure was performed for each extracted environmental factor.

### Determining the variables from the geocoding services

We checked environmental factors identified within the literature search regarding mapping possibilities with variables from Google Maps and OSM. Google Maps data, among other sources, are derived from official registries, e.g. from the Agency for Digitisation, High-Speed Internet and Surveying in Bavaria [26, 27]. OpenStreetMap, in contrast, is based on volunteered geographical information (VGI), i.e. it is based on user-generated content [28]. We have chosen both geocoding services because their data were freely available at low cost. Furthermore, their accuracy has been investigated for Germany in the past. Apparently, Google Maps showed higher completeness and higher precision of coordinates than OSM [17]. Besides environmental obesogenic factors, additional variables concerning the regional healthcare structure were taken into account. Four researchers in our team independently rated the relevance of the variables from the geocoding services with respect to the results of the literature search. After discussion, the variables best operationalizing the identified factors from the literature were determined and downloaded from Google Maps and OSM. We focused our analysis on single points of interest (POIs). Therefore, complex variable constructs, such as ‘neighbourhood walkability’ and ‘land use mix’, were not considered, as these compound measures are based e.g. on residential density or numbers of developed hectares which cannot be directly derived from online geocoding services. For an overview on the composites of these variables see Feng et al. [29]. Furthermore, six broader categories, ‘food’, ‘doctor’, ‘sport’, ‘education’, ‘transport’ and ‘other’, were determined via expert discussions within our team, and each operationalized variable, for which POIs were returned by at least one geocoding service, was assigned to one of those categories. Based on this approach, it was possible to visualize the distribution of environmental factors on a higher aggregated level and improve interpretability of the field validation results.

### Choosing locations for the validation process

We have chosen four pilot areas in the German federal state of Bavaria for the validation process. Our aim was to investigate the data quality of the geocoding services within regions of different population density and urbanization level. Size and population count of each area were derived from the German Federal Statistical Office and the statistical offices of the German Länder (federal states) [30]. The first region was a sparsely populated municipality in the south-west of Bavaria with fewer than 2000 inhabitants encompassing an area of about 36.31 km^2^. This area constituted a rural region containing few amenities (Area A). The second area was a street in a medium-sized major district town near Munich, the capital of Bavaria, with fewer than 45,000 inhabitants and a size of around 34.96 km^2^ (Area B). Finally, the densely populated city of Munich (about 1.5 million inhabitants, total area 310.71 km^2^) was selected for the validation. From the whole city of Munich, both a denser area close to the city centre and an area with a relatively lower density of amenities was chosen (Areas C and D).

### Database extraction and processing of data

**Fig. 2:**
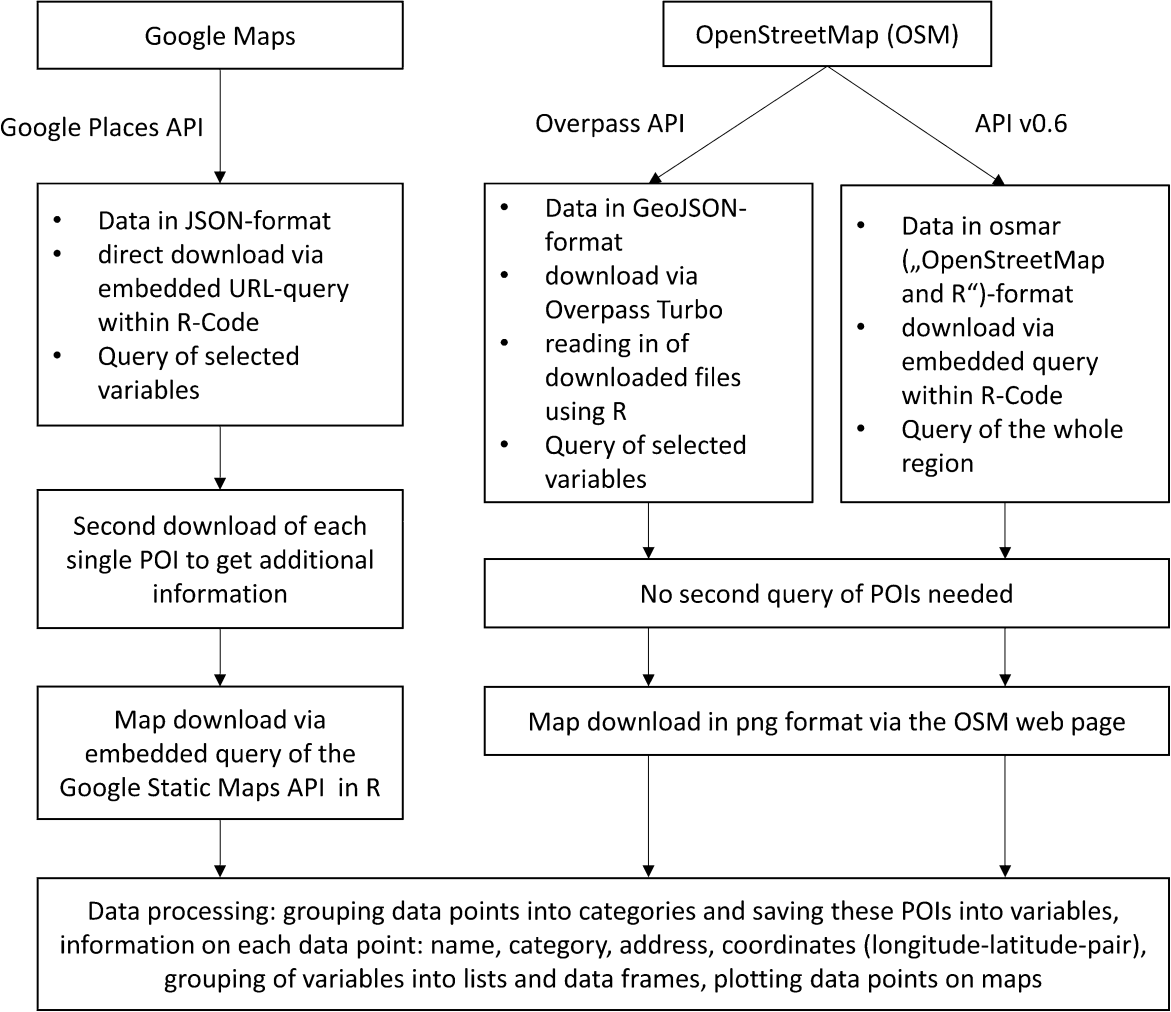
Overview on the data and query structure. *JSON* JavaScript Object Notation, *API* application programming interface, *POI* point of interest

**Fig. 3:**
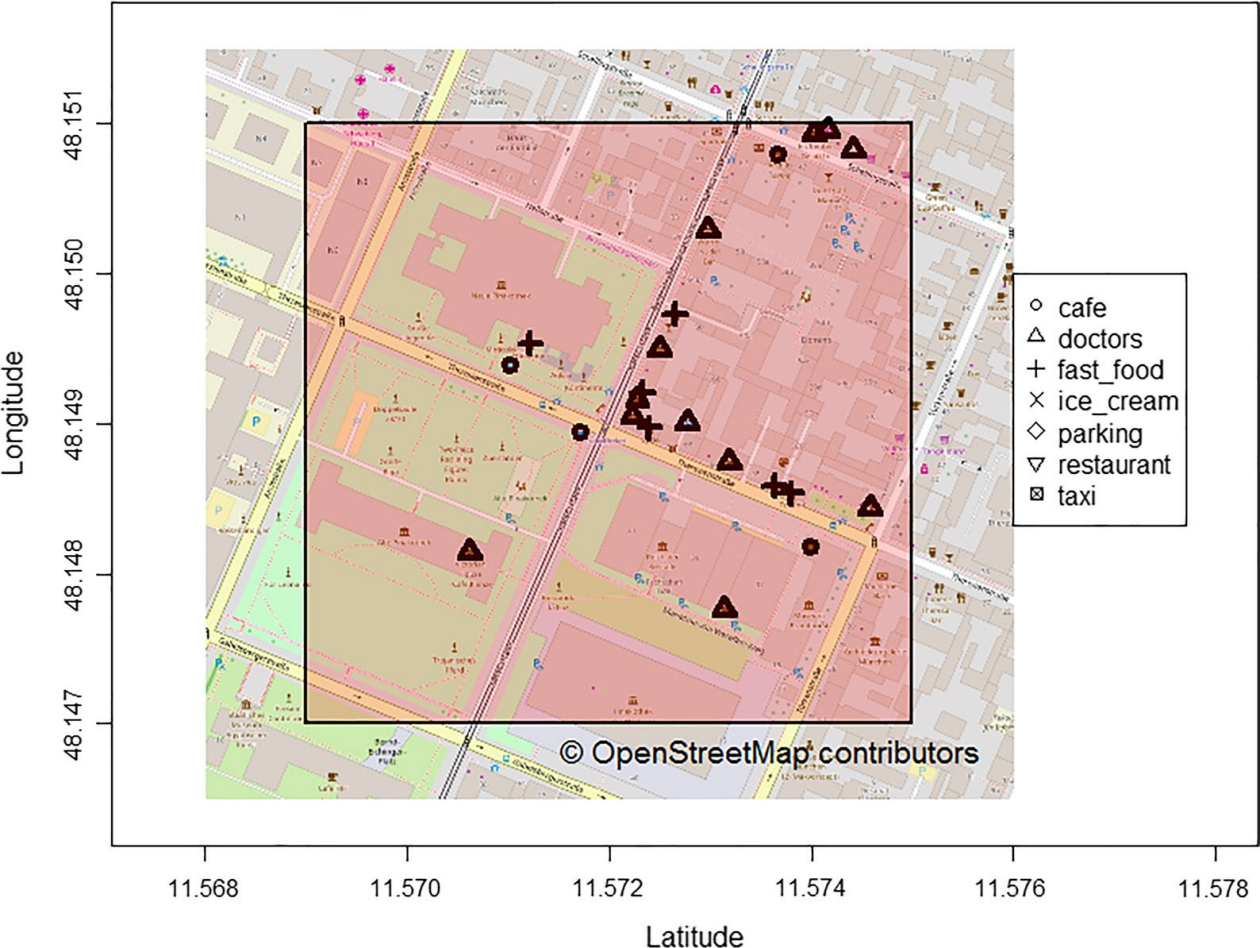
Example of visualization of OpenStreetMap data points (Area D)

Google Maps data were downloaded using queries in uniform resource locator (URL) format targeting the Google Places API. Furthermore, OSM queries were performed using a web interface and an OSM-based R package. The geographical database returns data in JavaScript Object Notation (JSON), GeoJSON or osmar (‘OpenStreetMap and R’) format, which are standard representations for geographical data. Based on the structure of these data formats, information for each of the single POIs can be accessed efficiently via a hierarchical structure and subsequently processed. For the Google Maps results, each entry had to be queried again in order to get additional relevant information, e.g. on names, addresses and categorizations. Using the downloaded OSM data, additional information could be extracted directly from the previously described data formats without any additional query. An overview of the data formats and query possibilities is shown in Fig. 2. The return of the spatial databases was checked regarding consistency and plausibility. Important examinations were identifying POIs that were counted twice or more because of being listed within different categories and checking whether the return of the database lies completely within the pre-specified search area. Additionally, spatial POIs were visualized on maps in order to check coherence. The geographical data points were marked according to their factor category, and the search area was also plotted. An example of visualization of some factors for Area D can be found in Fig. 3 for OSM. The underlying code and the other codes regarding Area D are available on github [https://github.com/MAPraeger/GOcode. Accessed 23 April 2019].

### Search area and download capacity

The shape of the downloaded regions was predefined by the geocoding services. OSM areas were rectangular, whereas Google Maps areas were circular. In order to make the shapes of OSM and Google Maps queries more comparable, we defined OSM search regions as quadratic. Further differences between the geocoding services affected the maximum downloadable data size. At the time of data download in 2017, Google Maps allowed up to 200 results per query and 1000 queries per day per person at zero costs [31], whereas OSM had fewer restrictions [32]. Depending on the API and the download tools used, areas of arbitrary size, whole so-called ‘planet files’ [33] or nearly arbitrary data sizes caused by memory overload within the statistical software, could be downloaded. Therefore, areas for the validation process were determined such that none of the above-mentioned restrictions took effect. Owing to the lower number of spatial POIs within the rural region (Area A), a wider area containing the whole municipality was chosen compared with the more urban areas (Areas B–D), for which the diameters of the circles and the edges of the squares were set to 200 metres.

### Validation process

Four researchers in our team locally scanned the predefined validation areas looking for the existence of the downloaded POIs of Google Maps and OSM. We designed a template to standardize the recording process and used maps containing the data points to improve efficiency. The number of returned POIs of a database was called ‘hits’. Each researcher documented the validation date, confirmation (true positive hit) or rejection (false positive hit) of existence of the POIs and new record of false negatives, i.e. data points discovered in the field that were not covered by Google Maps or OSM or both. After completion, the templates were digitalized.

If uncertainties regarding the existence of a POI were present during validation in the field, the researchers recorded their comments. If these notes indicated restrictions, e.g. regarding access to certain facilities during in-field validations, several online search engines were used to resolve these uncertainties. Further examples were incorrect categorization or implausible numbers of false positives at a certain place. To overcome these issues, we visited the home pages of the affected amenities and considered business directories (yellow pages).

Common summary statistics for the validation of geographical data points were calculated. For the quality assessment of the performance of a geocoding service for a given area, sensitivities, i.e. true positives divided by the sum of true positives and false negatives, and positive predictive values (PPVs), i.e. true positives divided by the sum of true positives and false positives, were calculated [34, 35].

### Software

We used the free software environment R, version 3.3.2, to implement code targeting the Google Places API via embedded URL query and for processing of the query results [36]. In order to download data from OSM, we applied an online tool for data filtering (Overpass Turbo) and the R package ‘osmar’ [37, 38]. For data processing, we used the packages ‘geojsonR’, ‘jsonlite’ and ‘rgdal’ and, for data visualization, the R packages ‘ggmap’ and ‘ggplot2’ [39–43].

## Results

### Literature search

An extensive list of environmental factors and the corresponding references (N = 256) can be found within Additional file 1: Table S1. The table contains the numbers of studies describing positive correlations, negative correlations and studies without significant associations for a given environmental factor. According to the definition of correlation within the methods section, overall positive correlations with weight status were discovered for the variables ‘fast food’, ‘food retail’, ‘unhealthy food outlets’, ‘convenience store’, ‘rural areas’, ‘urban sprawl’, ‘county sprawl’, ‘traffic’, ‘transport’ and ‘poverty’. Overall negative correlations were found for the variables ‘(healthy) food outlets’, ‘restaurants’, ‘supermarkets’, ‘tree cover’, ‘fitness or physical activity facilities’, ‘forests’, ‘greenspace’, ‘longer way to school’, ‘open space’, ‘outdoor recreation’, ‘park’, ‘recreation centre’, ‘walkability’, ‘aesthetics’, ‘intersection density’, ‘land use mix’, ‘population density’, ‘safety’, ‘sidewalk completeness’, ‘street connectivity’, ‘education’ and ‘physician supply’.

### Chosen variables from Google Maps and OSM

Tables 1 and 2 show the factors from Google Maps (N = 25 in total) and OSM (N = 126 in total) chosen for the validation process. Owing to the extent of the OSM variable pool, the relevant factors in the category ‘amenity’ are shown within Table 2 (N = 42). The full list of OSM variables is shown in Additional file 2: Table S2.

**Table 1:** Selected variables from the Google Maps pool

Bakery	Bar	Bus station	Cafe
Convenience store	Dentist	Doctor	Food
Grocery or supermarket	Gym	Hospital	Meal delivery
Meal takeaway	Park	Pharmacy	Physiotherapist
Restaurant	School	Spa	Stadium
Subway station	Taxi stand	Train station	Transit station
University

**Table 2:** Selected OpenStreetMap (OSM) variables in the category ‘amenity’

Bar	Bbq	Biergarten	Cafe	Fast food
Food court	Ice cream	Pub	Restaurant	College
School	Bicycle parking	Bicycle rental	Boat sharing	Bus station
Taxi	Clinic	Dentist	Doctors	Hospital
Nursing home	Pharmacy	Dive centre	Dojo	Ranger station
Beach resort	Dance	Fishing	Fitness centre	Garden
Golf course	Ice rink	Nature reserve	Park	Pitch
Playground	Sports centre	Stadium	Swimming area	Swimming pool
Track	Water park			

### Distribution of database results across categories

**Fig. 4:**
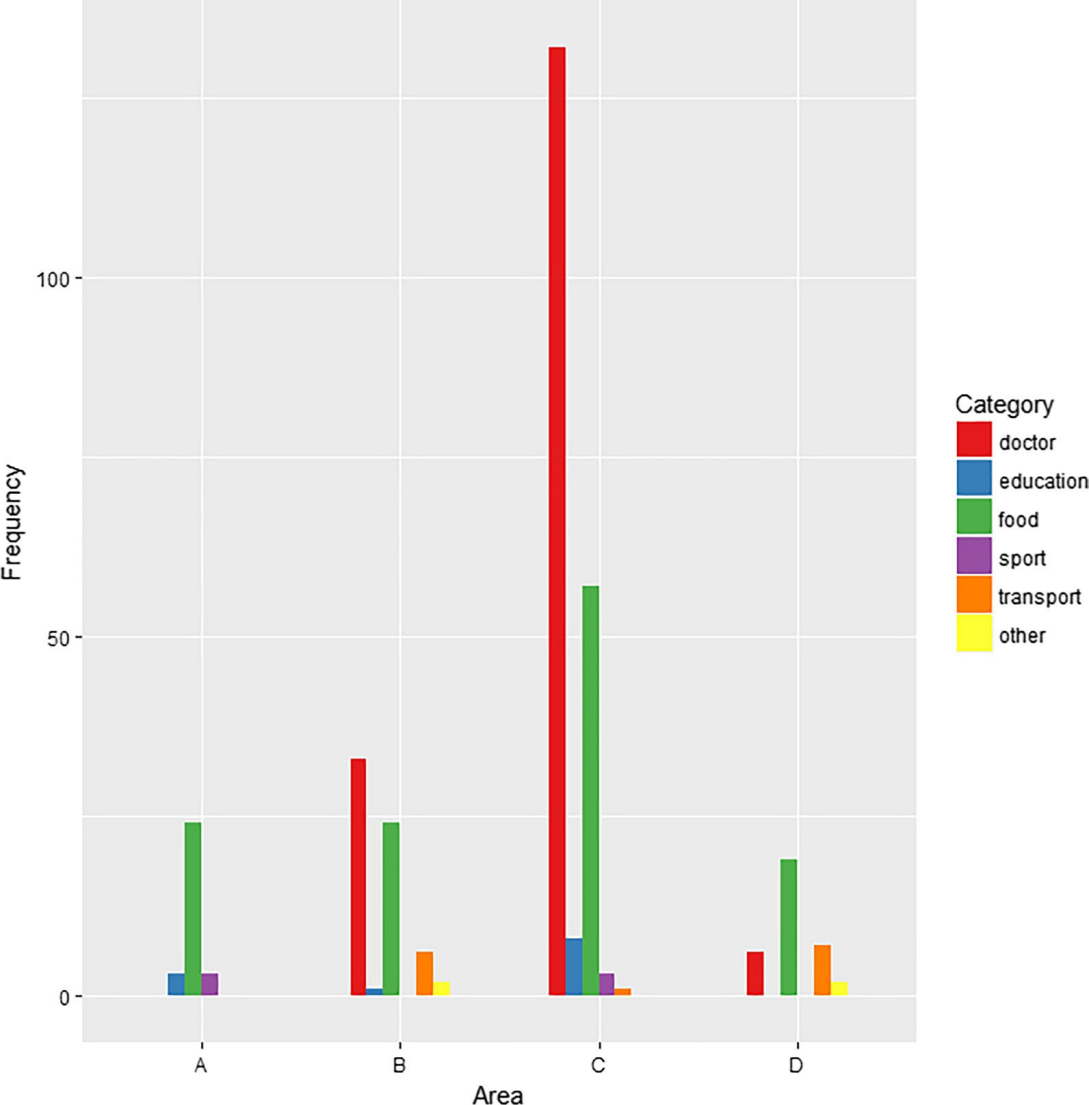
Distribution of hits across variable categories using Google Maps. Area A: sparsely populated municipality in the south-west of Bavaria. Area B: street in a medium-sized populated major district town near Munich. Area C: area close to the centre within the densely populated city of Munich. Area D: area with a lower density of amenities within the densely populated city of Munich

**Fig. 5:**
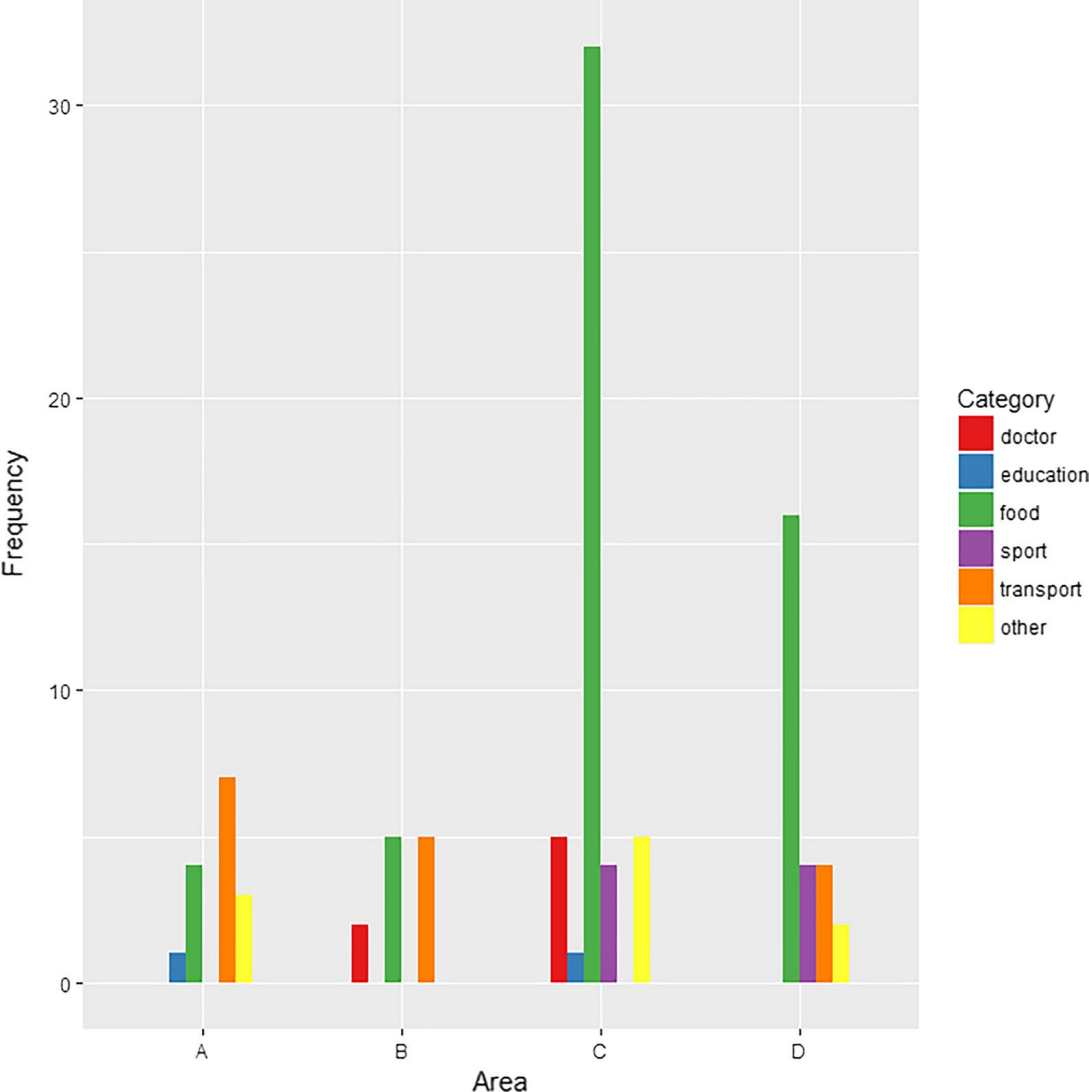
Distribution of hits across variable categories using OpenStreetMap. Area A: sparsely populated municipality in the south-west of Bavaria. Area B: street in a medium-sized populated major district town near Munich. Area C: area close to the centre within the densely populated city of Munich. Area D: area with a lower density of amenities within the densely populated city of Munich

Bar charts are shown for Google Maps (Fig. 4) and OSM (Fig. 5) in order to visualize the distribution of the database hits, i.e. the distribution of the sum of true-positive and false-positive entries of the geocoding services, across the six categories of ‘doctor’, ‘education’, ‘food’, ‘sport’, ‘transport’ and ‘other’ for the validation areas. Within the medium-sized populated Area B and the densely populated Area C, predominantly entries in the categories ‘doctor’ and ‘food’ account for most of the database hits in Google Maps. For the remaining areas, using Google Maps, ‘food’ was the most relevant category. Regarding OSM, the category ‘food’ was the most frequent category within Areas C and D of the city of Munich.

### Validations

Tables 3 and 4 show the numbers of true positives and false positives, PPVs, numbers of false negatives and sensitivity values for each validation area. As shown in the table, absolute numbers of true hits were higher for Google Maps than the corresponding numbers for OSM, irrespective of the validation area under consideration. Furthermore, false positives were also higher for Google Maps compared with OSM. The PPVs of OSM hits, ranging between 81 and 100%, were higher than the PPVs of Google Maps hits, which were found to be between 65 and 89%. In contrast, sensitivities were higher in Google Maps (between 59 and 98%) than in OSM (between 20 and 64%). False negatives were higher for OSM within three of the four validation areas. An overall comparison between the four areas showed that Area C within the city of Munich had the highest numbers of false negatives for both geocoding services. For OSM, high numbers of false negatives were also discovered for Area B, i.e. for the major district town. Predominantly during the validation within Area C, it became evident that the data quality regarding the variable category ‘doctor’ had a fundamental influence on the validation results. Therefore, we recalculated Table 3 without the POIs belonging to this category. The results of this recalculation process can be found within Table 4. Having omitted the category ‘doctor’, sensitivities of OSM improved for Area B and Area C. Within Area D, sensitivities of OSM were higher than sensitivities of Google Maps.

**Table 3:** Results of the field validation

Area	Geocoding service	True positives: N (% positive)^a^	False positives: N (% positive)	False negatives: N	Sensitivity^b^: %
A	Google Maps	19 (63.33)	11 (36.67)	13	59.38
A	OpenStreetMap	15 (88.24)	2 (11.76)	17	46.88
B	Google Maps	58 (89.23)	7 (10.77)	1	98.31
B	OpenStreetMap	12 (100)	0 (0)	47	20.34
C	Google Maps	144 (71.64)	57 (28.36)	63	69.57
C	OpenStreetMap	41 (87.23)	6 (12.77)	166	19.81
D	Google Maps	22 (64.71)	12 (35.29)	11	66.67
D	OpenStreetMap	21 (80.77)	5 (19.23)	12	63.64

Area A: sparsely populated municipality in the south-west of Bavaria

Area B: area in a medium-sized populated major district town near Munich

Area C: area close to the centre within the densely populated city of Munich

Area D: area with a lower density of amenities within the densely populated city of Munich

^a^The percentage of true positives is the positive predictive value (PPV) [PPV = true positives/(true positives + false positives)]

^b^Sensitivity = true positives/(true positives + false negatives)

**Table 4:** Results of the field validation without the category ‘doctor’

Area	Geocoding service	True positives: N (% positive)^a^	False positives: N (% positive)	False negatives: N	Sensitivity^b^: %
A	Google Maps	18 (62.07)	11 (37.93)	13	58.06
A	OpenStreetMap	15 (88.24)	2 (11.76)	16	48.39
B	Google Maps	29 (90.63)	3 (9.38)	1	96.67
B	OpenStreetMap	10 (100)	0 (0)	20	33.33
C	Google Maps	48 (69.57)	21 (30.43)	30	61.54
C	OpenStreetMap	36 (85.71)	6 (14.29)	42	46.15
D	Google Maps	19 (67.86)	9 (32.14)	6	76.00
D	OpenStreetMap	21 (80.77)	5 (19.23)	4	84.00

Area A: sparsely populated municipality in the south-west of Bavaria

Area B: area in a medium-sized populated major district town near Munich

Area C: area close to the centre within the densely populated city of Munich

Area D: area with a lower density of amenities within the densely populated city of Munich

^a^The percentage of true positives is the positive predictive value (PPV) [PPV = true positives/(true positives + false positives)]

^b^Sensitivity = true positives/(true positives + false negatives)

## Discussion

The aim of our study was to examine the feasibility of integrating information from online geocoding services for the assessment of environmental obesogenic factors that could potentially be used for diabetes surveillance. First, we identified variables correlated with obesogenic environments from the literature. Subsequently, we tested whether these variables could be reproduced using data from the online geocoding services Google Maps and OpenStreetMap (OSM). The results showed that this was possible given some restrictions, predominantly the diversity of the variable pools of the geocoding services and the complexity of the environmental factor to be projected. Maps created from the obesogenic and from protective data showed the geographical distribution of the environmental factors and were used within subsequent field validations. On the one hand, Google Maps showed greater completeness, i.e. lower proportion of false negatives, regarding POIs subsequently discovered in the field and the additional information assigned to them. Furthermore, the sensitivity of Google Maps was higher than the sensitivity of OSM. On the other hand, a higher PPV was seen for OSM in each of the validation areas.

Recently, the validity of the geocoding service Google Maps was tested using geoprocessing information [18]. Instead of using single geographical data points from the spatial databases of Google Maps and OSM, the authors compared virtual audit via Google Street View. Additionally, local field inspections were performed as the gold standard. It was shown that the validity and reliability of using Google Maps for the assessment of the built environment was high (Kappa of 78% and 80% respectively). Considering the German context, field inspections concerning the obesogenic environment have been performed in the past in order to record POIs [44]. Therefore, it was an important step within our study to inspect the database results of Google Maps and OSM locally.

PPVs of Google Maps and OSM found during our validation process were compared with each other. It became evident that the PPV of OSM was higher than the PPV for Google Maps in each region, because Google Maps showed considerably more false positives. Considering sensitivity, OSM showed lower values than Google Maps. Most influential variables regarding these comparisons were found within the category ‘doctor’. The data quality regarding physicians was better for Google Maps compared to OSM. Therefore, within areas with a higher share of doctors (Area B and Area C) the differences in sensitivities between Google Maps and OSM were large. Deleting the category ‘doctor’ from the analysis thus moderated this difference. False positives of Google Maps within the densely populated Area C were also mainly caused by the category ‘doctor’. The same category also contributed to the number of false negatives in OSM within this area and the sensitivity of OSM improved considerably after omitting POIs belonging to this category (see Table 4). To highlight the different influences of certain variable groups, it was an important step in our validation process to look for suitable stratification structures, such as the six categories ‘doctor’, ‘food’, ‘sport’, ‘transport’, ‘education’ and ‘other’.

In our study, we calculated the sensitivities and PPVs of Google Maps and OSM hits. They can be compared with the PPVs of other POI databases that we found in the existing literature. For example, Clary and colleagues [34] validated a Canadian food outlet database in the field. Comparing their database results with the actual occurrences in the field, the authors found sensitivities between 54.5 and 65.5% as well as PPVs between 64.4 and 77.3%. Within our study, the PPV for OSM was markedly higher (between 81 and 100%), whereas Google Maps had a more similar PPV compared with the Canadian database (between 63 and 89%). Regarding sensitivity, OSM showed lower values within three of the four validation areas (between 20 and 64%), whereas Google Maps sensitivities were at least comparable (between 59 and 98%) with the food outlet database.

To evaluate features of the obesogenic environment, Bethlehem and colleagues [14] performed a virtual audit based on Google Earth (GE) and Google Street View (GSV). They assessed the aspects walking, cycling, public transport, aesthetics, land use mix, grocery stores, food outlets and recreational facilities using observers. Virtual audit was found to be a valid and reliable approach. Within our study, we used Google Maps and OSM APIs for the programmed download of POIs, which does not need individual assessment for data collection.

Within our analyses, it also became evident that new variable entries appear more frequently, but old entries were deleted with time lag within the Google Maps database. The more specific variables in the OSM pool made it possible to identify some POIs that could not be precisely queried by Google. For example, OSM made it possible to extract ‘fast food’ instead of the broader category ‘food’. This feature nevertheless required taking into account all relevant specific factors describing a variable at a higher level in order to exhaust the OSM database completely.

### Strengths and limitations

Our study is based on an extensive literature search extracting factors of obesogenic environments. We used freely available data from global geocoding services Google Maps and OSM and applied various methods for downloading and processing geographical data using new query codes in the R programming environment. Finally, we validated our results with in-field inspections. To evaluate both physical activity and food-related environmental factors, composite approaches are required, which have been performed rather infrequently in the past [12]. Within our approach, we combined the food environment and the physical activity environment into a single layer containing POIs of the obesogenic factors and POIs of the protective factors.

Some limitations of our study have to be mentioned. First, it is focused on evidence from the literature based on an energy imbalance model [45]. However, according to the recent literature, other etiological causes for the development of obesity have to be considered to fully understand the underlying mechanisms, e.g. the carbohydrate-insulin model of obesity (beyond ‘calories in, calories out’) [46] or dietary behaviour (‘ultra-processed food vs unprocessed food’) [47]. Second, the literature search had a broad scope by updating and complementing a systematic review; however, a large number of the identified studies originated from the US. Structural differences regarding the built environment in US and European cities may influence direct transferability to the European context. For example, cities in the US are much more car dependent than European cities, which results in expected different health effects of environmental factors associated with physical activity [48]. Furthermore, instead of unhealthy corner stores in the US, in European countries, healthy stores selling fresh fruit and vegetables exist more often and are more evenly distributed across the cities [49]. A third drawback regarding our literature search could be publication bias, which would influence the assessment of the overall correlation of an environmental factor [50, 51]. Fourth, a significant proportion of the environmental factors was not correlated with obesity in the same direction across studies. Given this restriction, we have summarized the correlations found in the literature based on expert decision. Fifth, the precision and feasibility of variable extraction fundamentally depend on the variable pool structure of the geocoding service. Differences in the definition of a variable across geocoding services hamper direct comparisons of variables. Within our study, we found that the variable pool of OSM contains many more variables than Google Maps for a large number of environmental factors. Finding broader categories for environmental factors within our analysis made it easier to compare variables across geocoding services. Sixth, our study was limited to a German environment; therefore, generalization of our findings needs further assessment in other countries. Seventh, we have downloaded spatial POIs at a certain point in time; thus, we cannot make inferences on time effects. However, this cross-section offers an important starting point for future analyses. Eighth, each geographical area was validated by a different researcher; therefore, interobserver variability could have appeared during validation. In order to counteract this kind of bias, prior instructions were defined as precisely as possible, and discussions between the observers took place both before and after the validations. Finally, some restrictions regarding access to certain facilities appeared during validations in the field, mostly concerning database hits of the category ‘doctor’. Results of the validation process without this category are shown in Table 4. Within the analysis including the category ‘doctor’, this generated some uncertainties; therefore, we used the best available evidence, i.e. the home pages of these amenities and business directories (yellow pages). However, these uncertainties occurred only in a small number of cases and were discussed in detail during processing of the validation results.

The aim of our study was to examine the feasibility of using data from online geocoding services for diabetes surveillance. We were able to integrate information from these services by downloading, processing and visualizing their data on maps. The reliability of these variables was assessed within field validations and by search engines. Future examinations could test further types of variables from other research areas.

## Conclusions

Based on an extensive literature search, environmental factors could be identified that are associated with obesity. These factors could be partly operationalized through the variables and data available from the online geocoding services Google Maps and OSM. Using APIs, spatial data points could be identified and subsequently visualized on maps. Our findings showed that the validity of data from online geocoding services was reasonable. Consequently, environmental obesogenic factors could be described with our methodology and potentially used within diabetes surveillance. Further validation studies are needed to investigate the importance of environmental obesogenic factors.

## Additional files

**Additional file 1: Table S1.** Factors determined by literature search.

**Additional file 2: Table S2.** Complete list of chosen OSM variables.

## Data Availability

The data generated for this study were downloaded from Google Maps and OSM and the exemplary code is available on github [https://github.com/MAPraeger/GOcode. Accessed 23 April 2019] as described in the methods section.

## Abbreviations

API: application programming interface
BMI: body mass index
GE: Google Earth
GIS: geographic information system
GSV: Google Street View
JSON: JavaScript Object Notation
OSM: OpenStreetMap
POI: point of interest
PPV: positive predictive value
T2DM: type 2 diabetes mellitus
URL: uniform resource locator
VGI: volunteered geographical information
