# Supplementary material for: Using data from online geocoding services for the assessment of environmental obesogenic factors: a feasibility study
Source: Int J Health Geogr. 2019 Jun 7;18:13. doi: 10.1186/s12942-019-0177-9 (PMC6555943; doi:10.1186/s12942-019-0177-9)
Supplement: Supplementary file 1 — Additional file 1: Table S1. Factors determined by literature search. [file 12942_2019_177_MOESM1_ESM.doc]

Additional file 1

Table S1: Factors determined by literature search

| Factor | Google Maps | OpenStreetMap | Country | Negative correlation | Positive correlation | No association |
| --- | --- | --- | --- | --- | --- | --- |
| Factors regarding the food environment | | | | | | |
| (Healthy) Food outlets, healthy food | food, health | "amenity"="restaurant", "shop"="farm", "shop"="greengrocer", "shop"="supermarket" | USA, UK, Australia | [1-8] | [9] | [10] |
| Convenience store | convenience_store | "shop"="convenience" | USA, UK, Japan, Canada | [3, 11-14] | [15-24] | [11, 25-27] |
| Farmer markets | --- | "amenity"="marketplace", "shop"="farm" | USA | --- | [18] | --- |
| Fast food | food, meal_delivery, meal_takeaway | "amenity"="fast_food" | USA, New Zealand, UK, Japan, Canada, China, Germany | [3, 16, 28-32] | [12, 17, 20, 23, 25, 33-57] | [4, 13, 14, 26, 43, 54, 58-64] |
| Food advertising | --- | --- | USA | --- | [65] | --- |
| Food basket price / Relative food prices | --- | --- | USA | [66, 67] | --- | --- |
| Food retail | food | "amenity"="fast_food", "amenity"="restaurant", "shop"="convenience", "shop"="farm", "shop"="greengrocer", "shop"="mall", "shop"="supermarket" | USA, Canada, Australia, Ghana | [68] | [9, 31, 69-71] | [72] |
| Grocery stores | grocery_or_supermarket | "shop"="greengrocer" | USA, UK, China | [14, 24, 30, 73-75] | [16, 25, 28, 35, 71, 72, 75-77] | [29, 77-80] |
| Healthy and unhealthy food | health | "amenity"="bbq", "amenity"="fast_food", "shop"="convenience", "shop"="greengrocer" | USA | [81] | --- | --- |
| Nutrition environments (high/low) | bakery, bar, food | "amenity"="bbq", "amenity"="food_court", "amenity"="ice_cream", "craft"="bakery", "craft"="caterer", "shop"="bakery", "shop"="beverages", "shop"="butcher", "shop"="cheese", "shop"="chocolate", "shop"="coffee", "shop"="confectionery", "shop"="convenience", "shop"="convenience", "shop"="dairy", "shop"="deli", "shop"="farm", "shop"="ice_cream", "shop"="nutrition_supplements", "shop"="pasta", "shop"="pastry", "shop"="seafood", "shop"="spices", "shop"="tea", "shop"="wine", "vending"="bread", "amenity"="fast_food", "shop"="greengrocer" | USA | [82, 83] | --- | --- |
| Other food stores | convenience_store, grocery_or_supermarket | "amenity"="fast_food", "amenity"="restaurant", "shop"="convenience", "shop"="farm", "shop"="greengrocer", "shop"="mall", "shop"="supermarket" | UK, France | [24, 84] | [24, 85] | --- |
| Prepared food sites | food, meal_delivery, meal_takeaway | "amenity"="fast_food" | USA | --- | [86, 87] | --- |
| Quality and availability of daily shopping | shopping_mall | "shop"="beverages", "shop"="bicycle", "shop"="butcher", "shop"="cheese", "shop"="chocolate", "shop"="coffee", "shop"="confectionery", "shop"="convenience", "shop"="dairy", "shop"="deli", "shop"="farm", "shop"="fishing", "shop"="free_flying", "shop"="garden_centre", "shop"="garden_furniture", "shop"="greengrocer", "shop"="hunting", "shop"="ice_cream", "shop"="medical_supply", "shop"="nutrition_supplements", "shop"="outdoor", "shop"="pasta", "shop"="pastry", "shop"="scuba_diving", "shop"="seafood", "shop"="spices", "shop"="sports", "shop"="supermarket", "shop"="swimming_pool", "shop"="tea", "shop"="wine","shop"="bakery" | Netherlands | [88] | --- | --- |
| Restaurants | food, restaurant | "amenity"="biergarten", "amenity"="restaurant" | USA, China, Canada | [11, 14, 20, 28, 29, 39-41, 43, 48, 51, 73, 75, 89] | [42] | [11, 25, 27, 43, 90] |
| Side walk cafes | bar | "amenity"="bar", "amenity"="cafe", "amenity"="pub" | USA | [89, 91] | --- | --- |
| Supermarkets | convenience_store, grocery_or_supermarket | "shop"="mall", "shop"="supermarket" | USA, Japan, UK, Australia, Canada, France, Portugal | [4, 21, 26, 29, 30, 35, 58, 60, 92-98] | [17, 27, 71, 99] | [13, 100-107] |
| Unhealthy food outlets | food | "amenity"="fast_food" | UK, USA | --- | [1, 3, 8] | [7] |
| Factors regarding the physical activity environment | | | | | | |
| (Open) tree cover | --- | "natural"="tree", "natural"="tree_row", "natural"="wood" | USA | [91, 108, 109] | --- | --- |
| Access to quality parks (larger) | park | "boundary"="national_park", "leisure"="park" | USA | [110] | --- | --- |
| Built environment pattern | park | "boundary"="national_park", "highway"="cycleway", "leisure"="park","natural"="wood" | France | --- | --- | [111] |
| Automobile dependency, commuting time | --- | --- | USA | --- | [112] | --- |
| Bikeability | --- | "amenity"="bicycle_rental", "bicycle_road"="yes", "highway"="cycleway" | USA, USA | [113, 114] | --- | [115] |
| Coastline, access to the beach | --- | "natural"="coastline" | Australia, New Zealand | [116, 117] | --- | --- |
| Commuting time | --- | --- | USA | --- | [112, 118] | --- |
| Fitness facilities, physical activity facilities, sports facilities | gym, spa | "amenity"="dive_centre", "amenity"="dojo", "leisure"="dance", "leisure"="golf_course", "leisure"="ice_rink", "leisure"="pitch", "leisure"="swimming_area", "leisure"="swimming_pool", "leisure"="track", "sport", "leisure"="fitness_centre", "leisure"="sports_centre", "route"="fitness_trail", "route"="hiking", "route"="running" | USA, Australia, France, Spain, China, Canada | [29, 58, 64, 84, 86, 115, 116, 119-127] | [128, 129] | [27, 90, 94] |
| Forests, access to forests | --- | "natural"="wood" | USA | [108, 130, 131] | --- | --- |
| General physical activity level increase, no direct environmental factor | --- | --- | Canada | [132] | --- | --- |
| Greenness, green space | amusement_park, park | "boundary"="national_park", "leisure"="garden", "leisure"="nature_reserve", "leisure"="park", "natural"="fell", "natural"="grassland", "natural"="scrub", "natural"="tree", "natural"="tree_row", "natural"="wood" | USA, New Zealand, Europe (France, Germany, Slovakia, Hungary, Portugal, Italy, Switzerland, Lithuania), UK, Denmark, Canada, Australia, Netherlands, Finland, Germany | [88, 92, 96, 133-147] | [86, 141, 148-150] | [94, 133, 134, 143, 148, 149, 151-154] |
| Longer way (distance) to school | school, university | "amenity"="school","building"="school", | Spain, USA | [49, 50, 129] | --- | --- |
| NDVI (normalized difference vegetation index), only another (objective) measure of greenness | park | "natural" | USA | [145] | --- | --- |
| Negative perceptions of neighbourhood: crime, traffic, cleanness | --- | --- | Canada, USA, | --- | [155, 156] | --- |
| Neighbourhood activity supportiveness (residential density, number of parks, land-use mix, intersection density) | bicycle_store, bowling_alley, gym, park | "building"="…", "boundary"="national_park", "highway"="…", "landuse"=…", "leisure"="park", "store"="…" | USA | [157] | --- | --- |
| Open space | natural_feature | "landuse"="recreation_ground" | USA, China | [49, 50, 128] | [150] | --- |
| Outdoor recreation | amusement_park, rv_park, spa, zoo | "boundary"="national_park", "highway"="cycleway", "highway"="footway", "highway"="path", "highway"="rest_area", "leisure"="beach_resort", "leisure"="nature_reserve", "leisure"="park", "leisure"="playground", "leisure"="water_park", "natural"="water", "natural"="wood", "route"="fitness_trail", "route"="hiking", "route"="running", "tourism"="aquarium", "tourism"="camp_site", "tourism"="picnic_site", "tourism"="theme_park" | Canada, USA, Sweden, Australia | [27, 89, 115, 158, 159] | --- | [132, 160, 161] |
| Park characteristics, park land area | amusement_park, park | "boundary"="national_park", "leisure"="dog_park", "leisure"="park", "natural"="wood" | USA, UK, Chile, Canada, China, Australia | [22, 27, 30, 57, 110, 121, 134, 137, 162-167] | [23, 150] | [23, 152] |
| Parking quality and availability | parking | "amenity"="bicycle_parking", "amenity"="motorcycle_parking", "amenity"="parking" | Netherlands | --- | [88] | --- |
| Physical activity environment (high / low) | gym | "boundary"="national_park", "highway"="cycleway", "highway"="footway", "highway"="path", "leisure"="park", "natural"="wood", "route"="fitness_trail", "route"="hiking", "route"="running" | USA | [83, 168] | --- | --- |
| Playground | --- | "leisure"="playground" | USA | --- | --- | [59] |
| Recreation centres, recreation facilities | beauty_salon, gym, spa | "amenity"="kneipp_water_cure", "leisure"="fitness_centre", "leisure"="sports_centre" | USA, UK, Australia | [6, 12, 169-171] | --- | --- |
| River | --- | "amenity"="boat_sharing", "waterway" | China | [150] | --- | --- |
| Walkability | --- | "boundary"="national_park", "highway"="cycleway", "highway"="footway", "highway"="living_street", "highway"="path", "highway"="pedestrian", "leisure"="park", "natural"="wood" | USA, Canada, Australia, Belgium, Germany, France | [6, 37, 68, 113, 114, 132, 161, 172-194] | [37, 160, 195] | [57, 190, 193, 196-199] |
| Well-connected landscape | intersection | --- | USA | [108] | --- | --- |
| Factors regarding the urban form | | | | | | |
| (More) rural areas | --- | "boundaries", "place" | USA, Finland, Canada, Australia, Italy | [200] | [72, 118, 124, 199, 201-204] | [64, 204-206] |
| Aesthetics | --- | --- | USA, Netherlands | [88, 91, 120, 144, 147, 207-209] | [91] | [89] |
| Bus stop density | bus_station | "amenity"="bus_station", "highway"="bus_stop", "public_transport"="station" | USA, UK | [210] | [211] | [171] |
| County sprawl | --- | "building"="commercial", "building"="residential", "landuse"="commercial", "landuse"="residential" | USA | --- | [39, 212-217] | [218] |
| Destination intensity, destination accessibility | --- | --- | Australia, New Zealand | [219, 220] | --- | --- |
| Graffiti | --- | --- | Europe (France, Germany, Slovakia, Hungary, Portugal, Italy, Switzerland, Lithuania) | --- | [135] | --- |
| Housing density, dwelling density | --- | "boundaries", "building", "place" | USA, Australia, New Zealand, UK | [220] | [221] | [68, 171, 222] |
| Incivilities (breakdown of social order) | --- | --- | USA | --- | [135, 208] | --- |
| Infrastructure | bus_station, car_rental, taxi_stand, train_station | "amenity"="bus_station", "amenity"="car_rental", "amenity"="car_sharing", "amenity"="taxi", "building"="train_station", "highway"="bus_stop", "public_transport"="station" | USA | --- | [223] | [120] |
| Intensity of development | --- | --- | USA | [179] | --- | --- |
| Intersection density | intersection | --- | USA, France | [30, 94, 115, 124, 161, 166, 224, 225] | [14, 72, 226, 227] | [226] |
| Land use (mix) | --- | "building"="commercial", "building"="residential", "landuse"="allotments", "landuse"="commercial", "landuse"="farmland", "landuse"="farmyard", "landuse"="forest", "landuse"="grass", "landuse"="greenfield", "landuse"="greenhouse_horticulture", "landuse"="meadow", "landuse"="orchard", "landuse"="plant_nursery", "landuse"="recreation_ground", "landuse"="residential", "landuse"="village_green", "landuse"="vineyard" | USA, Australia, UK, Canada, China, International (Australia, Belgium, Brazil, China, Colombia, Czech Republic, Denmark, Mexico, New Zealand, Spain, the UK and USA) | [7, 47, 68, 125, 150, 161, 210, 226, 228-231] | [232] | [220, 226] |
| Landmark buildings | church, museum | "historic" | USA | [91] | --- | --- |
| Natural amenities (access to open water, varied topography and mild climate) | natural_feature | "natural" | USA | [233] | --- | --- |
| Residential density, population density | --- | "building"="commercial", "building"="residential", "landuse"="commercial", "landuse"="residential" | USA, UK, Canada, New Zealand, France, Portugal | [7, 14, 30, 64, 94, 97, 161, 166, 168, 178, 189, 201, 210, 225, 230, 234-237] | [166] | [72, 90, 106, 226] |
| Route exposure characteristics | route | "route" | USA | --- | --- | [148] |
| Safety, trust, no crime | fire_station, police | "amenity"="fire_station", "amenity"="police", "amenity"="prison" | USA, International (Australia, Belgium, Brazil, China, Colombia, Czech Republic, Denmark, Mexico, New Zealand, Spain, the UK and USA), Spain, Portugal, Australia, France | [6, 23, 27, 89, 120, 125, 137, 151, 204, 228, 238-240] | [91, 94, 175] | [29, 37, 59, 90, 164, 223, 240, 241] |
| Side walk completeness | --- | "sidewalk"="both / left / right / no" | USA, UK, Australia | [120, 161, 169, 207, 209] | [211] | --- |
| Street connectivity, road density | --- | "highway"="motorway", "highway"="primary", "highway"="residential", "highway"="secondary", "highway"="tertiary", "highway"="trunk", "highway:attribute = noexit = yes" | USA, UK, Canada, Australia, New Zealand, France | [56, 134, 220, 225, 230, 234, 242] | --- | [57, 94, 164, 178, 201, 230, 243, 244] |
| Streetscape | --- | --- | New Zealand | [220] | --- | --- |
| Subway station density, railway | subway_station, train_station | "public_transport" | USA | [50, 226] | --- | [226] |
| Traffic | airport, bus_station, car_rental, taxi_stand, train_station | --- | Canada, USA, Germany | [88, 161] | [143, 156, 209, 228, 245, 246] | [30, 143] |
| Transport | airport, bus_station, car_rental, taxi_stand, train_station | "aeroway"="aerodrome", "aeroway"="heliport", "amenity"="bus_station", "amenity"="car_rental", "amenity"="car_sharing", "amenity"="taxi", "building"="train_station", "highway"="bus_stop", "public_transport"="station", "railway"="station" | USA | [72, 225] | [176, 225, 229, 236] | --- |
| Urban sprawl | --- | "building"="commercial", "building"="residential", "landuse"="commercial", "landuse"="residential" | USA, Canada, Australia, International (Australia, Belgium, Brazil, China, Colombia, Czech Republic, Denmark, Mexico, New Zealand, Spain, the UK and USA) | --- | [222, 228, 247-249] | [214, 218, 222] |
| Other factors | | | | | | |
| Education | school, university | "amenity"="college", "amenity"="school" | USA, Canada, Finland, Portugal | [20, 30, 33, 37, 56, 64, 102, 106, 170, 202, 222, 248, 250-253] | --- | [72, 216] |
| Immigration | --- | --- | Canada | [222] | --- | [222] |
| Poverty | --- | --- | USA | --- | [14, 37, 39, 56, 57, 183, 187, 248, 253] | [37, 254, 255] |
| (Primary care) physician supply | doctor | "amenity" = "doctors" | USA | [64, 170, 256] | --- | --- |

References

1. Cetateanu A, Jones A: **Understanding the relationship between food environments, deprivation and childhood overweight and obesity: evidence from a cross sectional England-wide study**. *Health & place* 2014, **27**:68-76.

2. Hosler AS: **Retail food availability, obesity, and cigarette smoking in rural communities**. *The Journal of rural health : official journal of the American Rural Health Association and the National Rural Health Care Association* 2009, **25**(2):203-210.

3. Jennings A, Welch A, Jones AP, Harrison F, Bentham G, van Sluijs EM, Griffin SJ, Cassidy A: **Local food outlets, weight status, and dietary intake: associations in children aged 9-10 years**. *American journal of preventive medicine* 2011, **40**(4):405-410.

4. Larsen K, Cook B, Stone MR, Faulkner GEJ: **Food access and children's BMI in Toronto, Ontario: assessing how the food environment relates to overweight and obesity**. *International Journal of Public Health* 2015, **60**(1):69-77.

5. Miller LJ, Joyce S, Carter S, Yun G: **Associations between childhood obesity and the availability of food outlets in the local environment: a retrospective cross-sectional study**. *American journal of health promotion : AJHP* 2014, **28**(6):e137-145.

6. Moore K, Diez Roux AV, Auchincloss A, Evenson KR, Kaufman J, Mujahid M, Williams K: **Home and work neighbourhood environments in relation to body mass index: the Multi-Ethnic Study of Atherosclerosis (MESA)**. *Journal of epidemiology and community health* 2013, **67**(10):846-853.

7. Rundle A, Neckerman KM, Freeman L, Lovasi GS, Purciel M, Quinn J, Richards C, Sircar N, Weiss C: **Neighborhood food environment and walkability predict obesity in New York City**. *Environmental health perspectives* 2009, **117**(3):442-447.

8. Stark JH, Neckerman K, Lovasi GS, Konty K, Quinn J, Arno P, Viola D, Harris TG, Weiss CC, Bader MD *et al*: **Neighbourhood food environments and body mass index among New York City adults**. *Journal of epidemiology and community health* 2013, **67**(9):736-742.

9. Gantner LA, Olson CM, Frongillo EA: **Relationship of Food Availability and Accessibility to Women's Body Weights in Rural Upstate New York**. *Journal of Hunger and Environmental Nutrition* 2013, **8**(4):490-505.

10. Griffiths C, Frearson A, Taylor A, Radley D, Cooke C: **A cross sectional study investigating the association between exposure to food outlets and childhood obesity in Leeds, UK**. *International Journal of Behavioral Nutrition and Physical Activity* 2014, **11**.

11. Fan JX, Hanson HA, Zick CD, Brown BB, Kowaleski-Jones L, Smith KR: **Geographic scale matters in detecting the relationship between neighbourhood food environments and obesity risk: an analysis of driver license records in Salt Lake County, Utah**. *BMJ open* 2014, **4**(8):e005458.

12. Larson NI, Wall MM, Story MT, Neumark-Sztainer DR: **Home/family, peer, school, and neighborhood correlates of obesity in adolescents**. *Obesity (Silver Spring, Md)* 2013, **21**(9):1858-1869.

13. Mejia N, Lightstone AS, Basurto-Davila R, Morales DM, Sturm R: **Neighborhood Food Environment, Diet, and Obesity Among Los Angeles County Adults, 2011**. *Preventing chronic disease* 2015, **12**:E143.

14. Zick CD, Smith KR, Fan JX, Brown BB, Yamada I, Kowaleski-Jones L: **Running to the store? The relationship between neighborhood environments and the risk of obesity**. *Social science & medicine (1982)* 2009, **69**(10):1493-1500.

15. Berge JM, Wall M, Larson N, Forsyth A, Bauer KW, Neumark-Sztainer D: **Youth dietary intake and weight status: healthful neighborhood food environments enhance the protective role of supportive family home environments**. *Health & place* 2014, **26**:69-77.

16. Fraser LK, Edwards KL, Tominitz M, Clarke GP, Hill AJ: **Food outlet availability, deprivation and obesity in a multi-ethnic sample of pregnant women in Bradford, UK**. *Social science & medicine (1982)* 2012, **75**(6):1048-1056.

17. Hanibuchi T, Kondo K, Nakaya T, Nakade M, Ojima T, Hirai H, Kawachi I: **Neighborhood food environment and body mass index among Japanese older adults: results from the Aichi Gerontological Evaluation Study (AGES)**. *International journal of health geographics* 2011, **10**:43.

18. Jilcott SB, Wade S, McGuirt JT, Wu Q, Lazorick S, Moore JB: **The association between the food environment and weight status among eastern North Carolina youth**. *Public health nutrition* 2011, **14**(9):1610-1617.

19. Laska MN, Hearst MO, Forsyth A, Pasch KE, Lytle L: **Neighbourhood food environments: are they associated with adolescent dietary intake, food purchases and weight status?** *Public health nutrition* 2010, **13**(11):1757-1763.

20. Minaker LM, Raine KD, Wild TC, Nykiforuk CIJ, Thompson ME, Frank LD: **Objective Food Environments and Health Outcomes**. *American journal of preventive medicine* 2013, **45**(3):289-296.

21. Morland K, Diez Roux AV, Wing S: **Supermarkets, other food stores, and obesity: the atherosclerosis risk in communities study**. *American journal of preventive medicine* 2006, **30**.

22. Ohri-Vachaspati P, Lloyd K, Delia D, Tulloch D, Yedidia MJ: **A closer examination of the relationship between children's weight status and the food and physical activity environment**. *Preventive medicine* 2013, **57**(3):162-167.

23. Prince SA, Kristjansson EA, Russell K, Billette JM, Sawada MC, Ali A, Tremblay MS, Prud’homme D: **Relationships between neighborhoods, physical activity, and obesity: a multilevel analysis of a large Canadian City**. *Obes (Silver Spring)* 2012, **20**.

24. Yan R, Bastian ND, Griffin PM: **Association of food environment and food retailers with obesity in US adults**. *Health & place* 2015, **33**:19-24.

25. Block JP, Christakis NA, O'Malley AJ, Subramanian SV: **Proximity to food establishments and body mass index in the Framingham Heart Study offspring cohort over 30 years**. *American journal of epidemiology* 2011, **174**(10):1108-1114.

26. Bodor JN, Rice JC, Farley TA, Swalm CM, Rose D: **The association between obesity and urban food environments**. *Journal of urban health : bulletin of the New York Academy of Medicine* 2010, **87**(5):771-781.

27. Wall MM, Larson NI, Forsyth A, Van Riper DC, Graham DJ, Story MT, Neumark-Sztainer D: **Patterns of obesogenic neighborhood features and adolescent weight: a comparison of statistical approaches**. *American journal of preventive medicine* 2012, **42**(5):e65-75.

28. Ahern M, Brown C, Dukas S: **A National Study of the Association Between Food Environments and County‐Level Health Outcomes**. *The Journal of Rural Health* 2011, **27**(4):367-379.

29. Black JL, Macinko J, Dixon LB, Fryer GE, Jr.: **Neighborhoods and obesity in New York City**. *Health & place* 2010, **16**(3):489-499.

30. Huang R, Moudon AV, Cook AJ, Drewnowski A: **The spatial clustering of obesity: does the built environment matter?** *Journal of human nutrition and dietetics : the official journal of the British Dietetic Association* 2015, **28**(6):604-612.

31. Jilcott SB, McGuirt JT, Imai S, Evenson KR: **Measuring the retail food environment in rural and urban North Carolina counties**. *Journal of Public Health Management and Practice* 2010, **16**(5):432-440.

32. Pearce J, Hiscock R, Blakely T, Witten K: **A national study of the association between neighbourhood access to fast-food outlets and the diet and weight of local residents**. *Heal Place* 2009, **15**.

33. Burgoine T, Forouhi NG, Griffin SJ, Brage S, Wareham NJ, Monsivais P: **Does neighborhood fast-food outlet exposure amplify inequalities in diet and obesity? A cross-sectional study**. *The American journal of clinical nutrition* 2016, **103**(6):1540-1547.

34. Burgoine T, Forouhi NG, Griffin SJ, Wareham NJ, Monsivais P: **Associations between exposure to takeaway food outlets, takeaway food consumption, and body weight in Cambridgeshire, UK: population based, cross sectional study**. *BMJ (Clinical research ed)* 2014, **348**:g1464.

35. Chen H-J, Wang Y: **Changes in the neighborhood food store environment and children's body mass index at peripuberty in the United States**. *Journal of Adolescent Health* 2016, **58**(1):111-118.

36. Chen SE, Florax RJ, Snyder SD: **Obesity and fast food in urban markets: a new approach using geo-referenced micro data**. *Health economics* 2013, **22**(7):835-856.

37. Christman Z, Pruchno R, Cromley E, Wilson-Genderson M, Mir I: **A Spatial Analysis of Body Mass Index and Neighborhood Factors in Community-Dwelling Older Men and Women**. *International journal of aging & human development* 2016, **83**(1):3-25.

38. Fraser LK, Edwards KL: **The association between the geography of fast food outlets and childhood obesity rates in Leeds, UK**. *Health & place* 2010, **16**(6):1124-1128.

39. Gregson J: **Poverty, sprawl, and restaurant types influence body mass index of residents in California counties**. *Public health reports (Washington, DC : 1974)* 2011, **126 Suppl 1**:141-149.

40. Hollands S, Campbell MK, Gilliland J, Sarma S: **A spatial analysis of the association between restaurant density and body mass index in Canadian adults**. *Preventive medicine* 2013, **57**(4):258-264.

41. Hollands S, Campbell MK, Gilliland J, Sarma S: **Association between neighbourhood fast-food and full-service restaurant density and body mass index: a cross-sectional study of Canadian adults**. *Canadian journal of public health = Revue canadienne de sante publique* 2014, **105**(3):e172-178.

42. Inagami S, Cohen DA, Brown AF, Asch SM: **Body mass index, neighborhood fast food and restaurant concentration, and car ownership**. *Journal of urban health : bulletin of the New York Academy of Medicine* 2009, **86**.

43. Jeffery RW, Baxter J, McGuire M, Linde J: **Are fast food restaurants an environmental risk factor for obesity?** *Int J Behav Nutr Phys Act* 2006, **3**.

44. Kruger DJ, Greenberg E, Murphy JB, DiFazio LA, Youra KR: **Local concentration of fast-food outlets is associated with poor nutrition and obesity**. *American journal of health promotion : AJHP* 2014, **28**(5):340-343.

45. Lakes T, Burkart K: **Childhood overweight in Berlin: intra-urban differences and underlying influencing factors**. *International journal of health geographics* 2016, **15**.

46. Li F, Harmer P, Cardinal BJ, Bosworth M, Johnson-Shelton D: **Obesity and the built environment: does the density of neighborhood fast-food outlets matter?** *American journal of health promotion : AJHP* 2009, **23**(3):203-209.

47. Li F, Harmer PA, Cardinal BJ, Bosworth M, Acock A, Johnson-Shelton D, Moore JM: **Built environment, adiposity, and physical activity in adults aged 50-75**. *American journal of preventive medicine* 2008, **35**(1):38-46.

48. Mehta N, Chang VW: **Weight status and restaurant availability: a multilevel analysis**. *American journal of preventive medicine* 2008, **34**.

49. Oreskovic NM, Kuhlthau KA, Romm D, Perrin JM: **Built environment and weight disparities among children in high- and low-income towns**. *Academic pediatrics* 2009, **9**(5):315-321.

50. Oreskovic NM, Winickoff JP, Kuhlthau KA, Romm D, Perrin JM: **Obesity and the built environment among Massachusetts children**. *Clinical pediatrics* 2009, **48**(9):904-912.

51. Polsky JY, Moineddin R, Dunn JR, Glazier RH, Booth GL: **Absolute and relative densities of fast-food versus other restaurants in relation to weight status: Does restaurant mix matter?** *Preventive medicine* 2016, **82**:28-34.

52. Polsky JY, Moineddin R, Glazier RH, Dunn JR, Booth GL: **Local restaurant environment in relation to obesity among adults in Southern Ontario: Does restaurant mix matter?** *Canadian Journal of Diabetes* 2015, **39**:S25.

53. Pruchno R, Wilson-Genderson M, Gupta AK: **Neighborhood food environment and obesity in community-dwelling older adults: individual and neighborhood effects**. *American journal of public health* 2014, **104**(5):924-929.

54. Reitzel LR, Regan SD, Nguyen N, Cromley EK, Strong LL, Wetter DW, McNeill LH: **Density and proximity of fast food restaurants and body mass index among African Americans**. *American journal of public health* 2014, **104**(1):110-116.

55. Williams J, Scarborough P, Townsend N, Matthews A, Burgoine T, Mumtaz L, Rayner M: **Associations between Food Outlets around Schools and BMI among Primary Students in England: A Cross-Classified Multi-Level Analysis**. *PloS one* 2015, **10**(7):e0132930.

56. Xu Y, Wang F: **Built environment and obesity by urbanicity in the U.S**. *Health & place* 2015, **34**:19-29.

57. Xu Y, Wen M, Wang F: **Multilevel built environment features and individual odds of overweight and obesity in Utah**. *Applied geography (Sevenoaks, England)* 2015, **60**:197-203.

58. Boone-Heinonen J, Diez-Roux AV, Goff DC, Loria CM, Kiefe CI, Popkin BM, Gordon-Larsen P: **The neighborhood energy balance equation: does neighborhood food retail environment + physical activity environment = obesity? The CARDIA study**. *PloS one* 2013, **8**(12):e85141.

59. Burdette HL, Whitaker RC: **Neighborhood playgrounds, fast food restaurants, and crime: relationships to overweight in low-income preschool children**. *Preventive medicine* 2004, **38**(1):57-63.

60. Hattori A, An R, Sturm R: **Neighborhood food outlets, diet, and obesity among California adults, 2007 and 2009**. *Preventing chronic disease* 2013, **10**:E35.

61. Jiao J, Moudon AV, Kim SY, Hurvitz PM, Drewnowski A: **Health Implications of Adults' Eating at and Living near Fast Food or Quick Service Restaurants**. *Nutrition & Diabetes* 2015, **5**.

62. Mackenbach JD, Lakerveld J, Brug J, Nijpels G: **Understanding how access to fast food outlets is associated with obesity. The SPOTLIGHT project**. *Diabetologia* 2016, **59**(1 Supplement 1):S144.

63. Richardson AS, Meyer KA, Howard AG, Boone-Heinonen J, Popkin BM, Evenson KR, Shikany JM, Lewis CE, Gordon-Larsen P: **Multiple pathways from the neighborhood food environment to increased body mass index through dietary behaviors: A structural equation-based analysis in the CARDIA study**. *Health & place* 2015, **36**:74-87.

64. Slack T, Myers CA, Martin CK, Heymsfield SB: **The geographic concentration of US adult obesity prevalence and associated social, economic, and environmental factors**. *Obesity (Silver Spring, Md)* 2014, **22**(3):868-874.

65. Lesser LI, Zimmerman FJ, Cohen DA: **Outdoor advertising, obesity, and soda consumption: a cross-sectional study**. *BMC Public Health* 2013, **13**.

66. Lear SA, Gasevic D, Schuurman N: **Association of supermarket characteristics with the body mass index of their shoppers**. *Nutrition Journal* 2013, **12**.

67. Xu X, Variyam JN, Zhao Z, Chaloupka FJ: **Relative food prices and obesity in US Metropolitan areas: 1976-2001**. *PloS one* 2014, **9**(12):e114707.

68. Gebel K, Bauman AE, Sugiyama T, Owen N: **Mismatch between perceived and objectively assessed neighborhood walkability attributes: prospective relationships with walking and weight gain**. *Heal Place* 2011, **17**.

69. Dake FA, Thompson AL, Ng SW, Agyei-Mensah S, Codjoe SN: **The Local Food Environment and Body Mass Index among the Urban Poor in Accra, Ghana**. *Journal of urban health : bulletin of the New York Academy of Medicine* 2016, **93**(3):438-455.

70. Spence JC, Cutumisu N, Edwards J, Raine KD, Smoyer-Tomic K: **Relation between local food environments and obesity among adults**. *BMC Public Health* 2009, **9**.

71. Wang MC, Kim S, Gonzalez AA, MacLeod KE, Winkleby MA: **Socioeconomic and food-related physical characteristics of the neighbourhood environment are associated with body mass index**. *Journal of epidemiology and community health* 2007, **61**.

72. Yamada I, Brown BB, Smith KR, Zick CD, Kowaleski-Jones L, Fan JX: **Mixed land use and obesity: an empirical comparison of alternative land use measures and geographic scales**. *Prof Geogr* 2011, **64**.

73. Cerin E, Frank LD, Sallis JF, Saelens BE, Conway TL, Chapman JE, Glanz K: **From neighborhood design and food options to residents' weight status**. *Appetite* 2011, **56**(3):693-703.

74. Kapinos KA, Yakusheva O, Eisenberg D: **Obesogenic environmental influences on young adults: evidence from college dormitory assignments**. *Economics and human biology* 2014, **12**:98-109.

75. Zhang J, Wang HJ, Wang ZH, Zhang JG, Du WW, Su C, Jiang HR, Jia XF, Huang FF, Zhang B: **Influence of proximities to food outlets on daily energy intake among Chinese adults, evidence from china health and nutrition survey, 2009-2011**. *Obesity Reviews* 2016, **17**:159.

76. Gibson DM: **The neighborhood food environment and adult weight status: estimates from longitudinal data**. *American journal of public health* 2011, **101**(1):71-78.

77. Zenk SN, Mentz G, Schulz AJ, Johnson-Lawrence V, Gaines CR: **Longitudinal Associations Between Observed and Perceived Neighborhood Food Availability and Body Mass Index in a Multiethnic Urban Sample**. *Health education & behavior : the official publication of the Society for Public Health Education* 2017, **44**(1):41-51.

78. Gase LN, DeFosset AR, Smith LV, Kuo T: **The Association between Self-Reported Grocery Store Access, Fruit and Vegetable Intake, Sugar-Sweetened Beverage Consumption, and Obesity in a Racially Diverse, Low-Income Population**. *Frontiers in public health* 2014, **2**:229.

79. Mendez DD, Gary-Webb TL, Goode R, Zheng Y, Imes CC, Fabio A, Duell J, Burke LE: **Neighborhood factors and six-month weight change among overweight individuals in a weight loss intervention**. *Preventive medicine reports* 2016, **4**:569-573.

80. Tung EL, Peek ME, Makelarski JA, Lindau ST: **The built and social environment: Neighborhood obesity in vulnerable populations**. *Journal of General Internal Medicine* 2015, **30**:S267.

81. Hutchinson PL, Nicholas Bodor J, Swalm CM, Rice JC, Rose D: **Neighbourhood food environments and obesity in southeast Louisiana**. *Heal Place* 2012, **18**.

82. Paquet C, Coffee NT, Haren MT, Howard NJ, Adams RJ, Taylor AW, Daniel M: **Food environment, walkability, and public open spaces are associated with incident development of cardio-metabolic risk factors in a biomedical cohort**. *Health & place* 2014, **28**:173-176.

83. Saelens BE, Sallis JF, Frank LD, Couch SC, Zhou C, Colburn T, Cain KL, Chapman J, Glanz K: **Obesogenic neighborhood environments, child and parent obesity: the Neighborhood Impact on Kids study**. *American journal of preventive medicine* 2012, **42**(5):e57-64.

84. Casey R, Chaix B, Weber C, Schweitzer B, Charreire H, Salze P, Badariotti D, Banos A, Oppert J, Simon C: **Spatial accessibility to physical activity facilities and to food outlets and overweight in French youth**. *International journal of obesity* 2012, **36**(7):914-919.

85. Macdonald L, Ellaway A, Ball K, Macintyre S: **Is proximity to a food retail store associated with diet and BMI in Glasgow, Scotland?** *BMC Public Health* 2011, **11**.

86. Nies MA, Weber KT, Holmes J, Peterson T, Serr K, Arias J, Lim WY, Force R: **Spatial and Census Data to Evaluate Obese Persons and their Environment (SCOPE)**. *American journal of health behavior* 2015, **39**(4):582-588.

87. von Hippel P, Benson R: **Obesity and the natural environment across US counties**. *American journal of public health* 2014, **104**(7):1287-1293.

88. Putrik P, van Amelsvoort L, De Vries NK, Mujakovic S, Kunst AE, van Oers H, Jansen M, Kant I: **Neighborhood Environment is Associated with Overweight and Obesity, Particularly in Older Residents: Results from Cross-Sectional Study in Dutch Municipality**. *Journal of urban health : bulletin of the New York Academy of Medicine* 2015, **92**(6):1038-1051.

89. Drewnowski A, Aggarwal A, Rehm CD, Cohen-Cline H, Hurvitz PM, Moudon AV: **Environments perceived as obesogenic have lower residential property values**. *American journal of preventive medicine* 2014, **47**(3):260-274.

90. Zhao Z, Kaestner R, Xu X: **Spatial mobility and environmental effects on obesity**. *Economics and human biology* 2014, **14**:128-140.

91. Lovasi GS, Bader MD, Quinn J, Neckerman K, Weiss C, Rundle A: **Body mass index, safety hazards, and neighborhood attractiveness**. *American journal of preventive medicine* 2012, **43**(4):378-384.

92. Hsieh S, Klassen AC, Curriero FC, Caulfield LE, Cheskin LJ, Davis JN, Goran MI, Weigensberg MJ, Spruijt-Metz D: **Built environment associations with adiposity parameters among overweight and obese Hispanic youth**. *Preventive medicine reports* 2015, **2**:406-412.

93. Lamb KE, White SR: **Categorisation of built environment characteristics: the trouble with tertiles**. *Int J Behav Nutr Phys Act* 2015, **12**:19.

94. Leal C, Bean K, Thomas F, Chaix B: **Multicollinearity in associations between multiple environmental features and body weight and abdominal fat: using matching techniques to assess whether the associations are separable**. *American journal of epidemiology* 2012, **175**(11):1152-1162.

95. Li Y, Robinson LE, Carter WM, Gupta R: **Childhood obesity and community food environments in Alabama's Black Belt region**. *Child: care, health and development* 2015, **41**(5):668-676.

96. Liu GC, Wilson JS, Qi R, Ying J: **Green neighborhoods, food retail and childhood overweight: differences by population density**. *American journal of health promotion : AJHP* 2007, **21**(4 Suppl):317-325.

97. Lopez RP: **Neighborhood risk factors for obesity**. *Obesity (Silver Spring, Md)* 2007, **15**(8):2111-2119.

98. Viola D, Arno PS, Maroko AR, Schechter CB, Sohler N, Rundle A, Neckerman KM, Maantay J: **Overweight and obesity: can we reconcile evidence about supermarkets and fast food retailers for public health policy?** *Journal of public health policy* 2013, **34**(3):424-438.

99. Fiechtner L, Block J, Duncan DT, Gillman MW, Gortmaker SL, Melly SJ, Rifas-Shiman SL, Taveras EM: **Proximity to supermarkets associated with higher body mass index among overweight and obese preschool-age children**. *Preventive medicine* 2013, **56**(3-4):218-221.

100. Cummins S, Flint E, Matthews SA: **New neighborhood grocery store increased awareness of food access but did not alter dietary habits or obesity**. *Health affairs (Project Hope)* 2014, **33**(2):283-291.

101. Drewnowski A, Aggarwal A, Hurvitz PM, Monsivais P, Moudon AV: **Obesity and supermarket access: proximity or price?** *Am J Public Heal* 2012, **102**.

102. Drewnowski A, Moudon AV, Jiao J, Aggarwal A, Charreire H, Chaix B: **Food environment and socioeconomic status influence obesity rates in Seattle and in Paris**. *International journal of obesity (2005)* 2014, **38**(2):306-314.

103. Dubowitz T, Ghosh-Dastidar M, Cohen DA, Beckman R, Steiner ED, Hunter GP, Florez KR, Huang C, Vaughan CA, Sloan JC *et al*: **Diet And Perceptions Change With Supermarket Introduction In A Food Desert, But Not Because Of Supermarket Use**. *Health Affairs* 2015, **34**(11):1858-1868.

104. Dubowitz T, Zenk SN, Ghosh-Dastidar B, Cohen DA, Beckman R, Hunter G, Steiner ED, Collins RL: **Healthy food access for urban food desert residents: examination of the food environment, food purchasing practices, diet and BMI**. *Public health nutrition* 2015, **18**(12):2220-2230.

105. Hobbs M, Green M, Griffiths C, Jordan H, Saunders J, McKenna J: **How different data sources and definitions of neighbourhood influence the association between food outlet availability and body mass index: a cross-sectional study**. *Perspectives in public health* 2016.

106. Santana P, Santos R, Nogueira H: **The link between local environment and obesity: a multilevel analysis in the Lisbon Metropolitan Area, Portugal**. *Social science & medicine (1982)* 2009, **68**(4):601-609.

107. Zhang YT, Laraia BA, Mujahid MS, Blanchard SD, Warton EM, Moffet HH, Karter AJ: **Is a reduction in distance to nearest supermarket associated with BMI change among type 2 diabetes patients?** *Health & place* 2016, **40**:15-20.

108. Kim JH, Lee C, Olvara NE, Ellis CD: **The role of landscape spatial patterns on obesity in Hispanic children residing in inner-city neighborhoods**. *Journal of physical activity & health* 2014, **11**(8):1449-1457.

109. Ulmer JM, Wolf KL, Backman DR, Tretheway RL, Blain CJ, O'Neil-Dunne JP, Frank LD: **Multiple health benefits of urban tree canopy: The mounting evidence for a green prescription**. *Health & place* 2016, **42**:54-62.

110. Rundle A, Quinn J, Lovasi G, Bader MD, Yousefzadeh P, Weiss C, Neckerman K: **Associations between body mass index and park proximity, size, cleanliness and recreational facilities**. *Am J Heal Promot* 2013, **27**.

111. Charreire H, Weber C, Chaix B, Salze P, Casey R, Banos A, Badariotti D, Kesse-Guyot E, Hercberg S, Simon C: **Identifying built environmental patterns using cluster analysis and GIS: Relationships with walking, cycling and body mass index in French adults**. *International Journal of Behavioral Nutrition and Physical Activity* 2012, **9**(1):59.

112. Zhang XY, Holt JB, Lu H, Onufrak S, Yang JW, French SP, Sui DZ: **Neighborhood commuting environment and obesity in the United States: An urban-rural stratified multilevel analysis**. *Preventive medicine* 2014, **59**:31-36.

113. Brown BB, Smith KR, Hanson H, Fan JX, Kowaleski-Jones L, Zick CD: **Neighborhood design for walking and biking: physical activity and body mass index**. *American journal of preventive medicine* 2013, **44**.

114. Horacek TM, Dede Yildirim E, Kattelmann K, Brown O, Byrd-Bredbenner C, Colby S, Greene G, Hoerr S, Kidd T, Koenings MM *et al*: **Path Analysis of Campus Walkability/Bikeability and College Students' Physical Activity Attitudes, Behaviors, and Body Mass Index**. *American journal of health promotion : AJHP* 2016.

115. Timperio A, Jeffery RW, Crawford D, Roberts R, Giles-Corti B, Ball K: **Neighbourhood physical activity environments and adiposity in children and mothers: a three-year longitudinal study**. *Int J Behav Nutr Phys Act* 2010, **7**:18.

116. Abbott G, Backholer K, Peeters A, Thornton L, Crawford D, Ball K: **Explaining educational disparities in adiposity: the role of neighborhood environments**. *Obesity (Silver Spring, Md)* 2014, **22**(11):2413-2419.

117. Witten K, Hiscock R, Pearce J, Blakely T: **Neighbourhood access to open spaces and the physical activity of residents: a national study**. *Preventive medicine* 2008, **47**(3):299-303.

118. Kowaleski-Jones L, Wen M: **Community and child energy balance: differential associations between neighborhood environment and overweight risk by gender**. *International journal of environmental health research* 2013, **23**(5):434-445.

119. Black JL, Macinko J: **The changing distribution and determinants of obesity in the neighborhoods of New York City, 2003-2007**. *American journal of epidemiology* 2010, **171**(7):765-775.

120. Catlin TK, Simoes EJ, Brownson RC: **Environmental and policy factors associated with overweight among adults in Missouri**. *Am J Heal Promot* 2003, **17**.

121. Colabianchi N, Coulton CJ, Hibbert JD, McClure SM, Ievers-Landis CE, Davis EM: **Adolescent self-defined neighborhoods and activity spaces: spatial overlap and relations to physical activity and obesity**. *Health & place* 2014, **27**:22-29.

122. Ellaway A, Lamb KE, Ferguson NS, Ogilvie D: **Associations between access to recreational physical activity facilities and body mass index in Scottish adults**. *BMC Public Health* 2016, **16**:756.

123. Gordon-Larsen P, Nelson MC, Page P, Popkin BM: **Inequality in the built environment underlies key health disparities in physical activity and obesity**. *Pediatrics* 2006, **117**(2):417-424.

124. Hoehner CM, Allen P, Barlow CE, Marx CM, Brownson RC, Schootman M: **Understanding the independent and joint associations of the home and workplace built environments on cardiorespiratory fitness and body mass index**. *American journal of epidemiology* 2013, **178**(7):1094-1105.

125. Mobley LR, Root ED, Finkelstein EA, Khavjou O, Farris RP, Will JC: **Environment, obesity, and cardiovascular disease risk in low-income women**. *American journal of preventive medicine* 2006, **30**.

126. Troped PJ, Starnes HA, Puett RC, Tamura K, Cromley EK, James P, Ben-Joseph E, Melly SJ, Laden F: **Relationships between the built environment and walking and weight status among older women in three U.S. States**. *Journal of aging and physical activity* 2014, **22**(1):114-125.

127. Wilson DK, Ainsworth BE, Bowles H: **Body mass index and environmental supports for physical activity among active and inactive residents of a U. S. southeastern county**. *Heal Psychol* 2007, **26**.

128. Almeida FA, Wall SS, You W, Harden SM, Hill JL, Krippendorf BE, Estabrooks PA: **The association between worksite physical environment and employee nutrition, and physical activity behavior and weight status**. *Journal of occupational and environmental medicine* 2014, **56**(7):779-784.

129. Gutierrez-Zornoza M, Sanchez-Lopez M, Garcia-Hermoso A, Gonzalez-Garcia A, Chillon P, Martinez-Vizcaino V: **Active commuting to school, weight status, and cardiometabolic risk in children from rural areas: the Cuenca study**. *Health education & behavior : the official publication of the Society for Public Health Education* 2015, **42**(2):231-239.

130. Boncinelli F, Riccioli F, Marone E: **Do forests help to keep my body mass index low?** *Forest Policy and Economics* 2015, **54**:11-17.

131. Tsai WL, Floyd MF, Leung YF, McHale MR, Reich BJ: **Urban Vegetative Cover Fragmentation in the U.S.: Associations With Physical Activity and BMI**. *American journal of preventive medicine* 2016, **50**(4):509-517.

132. Berry TR, Spence JC, Blanchard CM, Cutumisu N, Edwards J, Selfridge G: **A longitudinal and cross-sectional examination of the relationship between reasons for choosing a neighbourhood, physical activity and body mass index**. *Int J Behav Nutr Phys Act* 2010, **7**:57.

133. Astell-Burt T, Feng X, Kolt GS: **Greener neighborhoods, slimmer people? Evidence from 246,920 Australians**. *International journal of obesity (2005)* 2014, **38**(1):156-159.

134. Coombes E, Jones AP, Hillsdon M: **The relationship of physical activity and overweight to objectively measured green space accessibility and use**. *Social science & medicine (1982)* 2010, **70**(6):816-822.

135. Ellaway A, Macintyre S, Bonnefoy X: **Graffiti, greenery, and obesity in adults: secondary analysis of European cross sectional survey**. *BMJ (Clinical research ed)* 2005, **331**.

136. Halonen JI, Kivimaki M, Pentti J, Stenholm S, Kawachi I, Subramanian SV, Vahtera J: **Green and blue areas as predictors of overweight and obesity in an 8-year follow-up study**. *Obesity (Silver Spring, Md)* 2014, **22**(8):1910-1917.

137. Mathis AL, Rooks RN, Tawk RH, Kruger DJ: **Neighborhood Influences and BMI in Urban Older Adults**. *Journal of applied gerontology : the official journal of the Southern Gerontological Society* 2015.

138. Nielsen TS, Hansen KB: **Do green areas affect health? Results from a Danish survey on the use of green areas and health indicators**. *Health & place* 2007, **13**.

139. Pearson AL, Bentham G, Day P, Kingham S: **Associations between neighbourhood environmental characteristics and obesity and related behaviours among adult New Zealanders**. *BMC Public Health* 2014, **14**:553.

140. Pereira G, Christian H, Foster S, Boruff BJ, Bull F, Knuiman M, Giles-Corti B: **The association between neighborhood greenness and weight status: an observational study in Perth Western Australia**. *Environmental health : a global access science source* 2013, **12**:49.

141. Prince SA, Kristjansson EA, Russell K, Billette J, Sawada M, Ali A, Tremblay MS, Prud D: **A multilevel analysis of neighbourhood built and social environments and adult self-reported physical activity and body mass index in Ottawa**. *Canada* 2011, **7600**.

142. Sanders T, Feng X, Fahey PP, Lonsdale C, Astell-Burt T: **Greener neighbourhoods, slimmer children? Evidence from 4423 participants aged 6 to 13 years in the Longitudinal Study of Australian children**. *International Journal of Obesity* 2015, **39**(8):1224-1229.

143. Schule SA, Fromme H, Bolte G: **Built and socioeconomic neighbourhood environments and overweight in preschool aged children. A multilevel study to disentangle individual and contextual relationships**. *Environmental research* 2016, **150**:328-336.

144. Stark JH, Neckerman K, Lovasi GS, Quinn J, Weiss CC, Bader MD, Konty K, Harris TG, Rundle A: **The impact of neighborhood park access and quality on body mass index among adults in New York City**. *Preventive medicine* 2014, **64**:63-68.

145. Tilt JH, Unfried TM, Roca B: **Using objective and subjective measures of neighborhood greenness and accessible destinations for understanding walking trips and BMI in Seattle.Washington**. *Am J Heal Promot* 2007, **21**.

146. Toftager M, Ekholm O, Schipperijn J, Stigsdotter U, Bentsen P, Gronbaek M, Randrup TB, Kamper-Jorgensen F: **Distance to green space and physical activity: a Danish national representative survey**. *J Phys Act Heal* 2011, **8**.

147. Van Der Zwaard BC, Schalkwijk A, Elders PJM, Nijpels G, Platt L: **Is access to the outdoors associated with childhood overweight and obesity?** *Diabetologia* 2015, **58**(1 SUPPL. 1):S94.

148. Burgoine T, Jones AP, Namenek Brouwer RJ, Benjamin Neelon SE: **Associations between BMI and home, school and route environmental exposures estimated using GPS and GIS: do we see evidence of selective daily mobility bias in children?** *International journal of health geographics* 2015, **14**:8.

149. Cummins S, Fagg J: **Does greener mean thinner? Associations between neighbourhood greenspace and weight status among adults in England**. *Int J Obes* 2012, **36**.

150. Ying Z, Ning LD, Xin L: **Relationship between built environment, physical activity, adiposity, and health in adults aged 46–80 in Shanghai, China**. *Journal of physical activity & health* 2015, **12**(4):569-578.

151. Lovasi GS, Schwartz-Soicher O, Quinn JW, Berger DK, Neckerman KM, Jaslow R, Lee KK, Rundle A: **Neighborhood safety and green space as predictors of obesity among preschool children from low-income families in New York City**. *Preventive medicine* 2013, **57**(3):189-193.

152. Potestio ML, Patel AB, Powell CD, McNeil DA, Jacobson RD, McLaren L: **Is there an association between spatial access to parks/green space and childhood overweight/obesity in Calgary, Canada?** *Int J Behav Nutr Phys Act* 2009, **6**:77.

153. Richardson EA, Pearce J, Mitchell R, Kingham S: **Role of physical activity in the relationship between urban green space and health**. *Public Health* 2013, **127**.

154. Ward JS, Duncan JS, Jarden A, Stewart T: **The impact of children's exposure to greenspace on physical activity, cognitive development, emotional wellbeing, and ability to appraise risk**. *Health & place* 2016, **40**:44-50.

155. Aggarwal A, Rehm C, Cohen-Cline H, Hurvitz P, Moudon A, Drewnowski A: **Residential property values: a novel GIS-based metric to characterize obesogenic environments**. *Faseb J* 2014, **28**(1):no pagination.

156. Berry TR, Spence JC, Blanchard C, Cutumisu N, Edwards J, Nykiforuk C: **Changes in BMI over 6 years: the role of demographic and neighborhood characteristics**. *International journal of obesity (2005)* 2010, **34**.

157. Carlson JA, Remigio-Baker RA, Anderson CA, Adams MA, Norman GJ, Kerr J, Criqui MH, Allison M: **Walking mediates associations between neighborhood activity supportiveness and BMI in the Women's Health Initiative San Diego cohort**. *Health & place* 2016, **38**:48-53.

158. Bjork J, Albin M, Grahn P, Jacobsson H, Ardo J, Wadbro J, Ostergren PO: **Recreational values of the natural environment in relation to neighbourhood satisfaction, physical activity, obesity and wellbeing**. *Journal of epidemiology and community health* 2008, **62**(4):e2.

159. Sullivan SM, Brashear MM, Broyles ST, Rung AL: **Neighborhood environments and obesity among Afro-Caribbean, African American, and Non-Hispanic white adults in the United States: results from the National Survey of American Life**. *Preventive medicine* 2014, **61**:1-5.

160. Adams MA, Todd M, Kurka J, Conway TL, Cain KL, Frank LD, Sallis JF: **Patterns of Walkability, Transit, and Recreation Environment for Physical Activity**. *American journal of preventive medicine* 2015, **49**(6):878-887.

161. Duncan DT, Sharifi M, Melly SJ, Marshall R, Sequist TD, Rifas-Shiman SL, Taveras EM: **Characteristics of walkable built environments and BMI z-scores in children: evidence from a large electronic health record database**. *Environmental health perspectives* 2014, **122**(12):1359-1365.

162. Armstrong B, Lim CS, Janicke DM: **Park Density Impacts Weight Change in a Behavioral Intervention for Overweight Rural Youth**. *Behavioral Medicine* 2015, **41**(3):123-130.

163. Mena C, Fuentes E, Ormazabal Y, Palomo-Velez G, Palomo I: **Role of access to parks and markets with anthropometric measurements, biological markers, and a healthy lifestyle**. *International journal of environmental health research* 2015, **25**(4):373-383.

164. Scott MM, Dubowitz T, Cohen DA: **Regional differences in walking frequency and BMI: what role does the built environment play for Blacks and Whites?** *Heal Place* 2009, **15**.

165. Veitch J, Abbott G, Kaczynski AT, Wilhelm Stanis SA, Besenyi GM, Lamb KE: **Park availability and physical activity, TV time, and overweight and obesity among women: Findings from Australia and the United States**. *Health & place* 2016, **38**:96-102.

166. Wen M, Kowaleski-Jones L: **The built environment and risk of obesity in the United States: racial-ethnic disparities**. *Health & place* 2012, **18**(6):1314-1322.

167. West ST, Shores KA, Mudd LM: **Association of Available Parkland, Physical Activity, and Overweight in America’s Largest Cities**. *J Public Heal Manag Pr* 2012, **18**.

168. Compernolle S, Oppert JM, Mackenbach JD, Lakerveld J, Charreire H, Glonti K, Bardos H, Rutter H, De Cocker K, Cardon G *et al*: **Mediating role of energy-balance related behaviors in the association of neighborhood socio-economic status and residential area density with BMI: The SPOTLIGHT study**. *Preventive medicine* 2016, **86**:84-91.

169. Giles-Corti B, Macintyre S, Clarkson JP, Pikora T, Donovan RJ: **Environmental and lifestyle factors associated with overweight and obesity in Perth, Australia**. *American journal of health promotion : AJHP* 2003, **18**(1):93-102.

170. Myers CA, Slack T, Martin CK, Broyles ST, Heymsfield SB: **Change in Obesity Prevalence across the United States Is Influenced by Recreational and Healthcare Contexts, Food Environments, and Hispanic Populations**. *PloS one* 2016, **11**(2):e0148394.

171. Sarkar C, Gallacher J, Webster C: **Built environment configuration and change in body mass index: the Caerphilly Prospective Study (CaPS)**. *Health & place* 2013, **19**:33-44.

172. Casagrande SS, Gittelsohn J, Zonderman AB, Evans MK, Gary-Webb TL: **Association of walkability with obesity in Baltimore City, Maryland**. *Am J Public Heal* 2011, **101**.

173. Chiu M, Shah B, Rezai MR, Austin P, Tu J: **Neighbourhood walkability and risk of obesity**. *Canadian Journal of Diabetes* 2014, **38**:S39.

174. Creatore MI, Glazier RH, Moineddin R, Fazli GS, Johns A, Gozdyra P, Matheson FI, Kaufman-Shriqui V, Rosella LC, Manuel DG *et al*: **Association of Neighborhood Walkability With Change in Overweight, Obesity, and Diabetes**. *Jama* 2016, **315**(20):2211-2220.

175. Doyle S, Kelly-schwartz A, Schlossberg M, Stockard J: **Active Community Environments and Health: The Relationship of Walkable and Safe Communities to Individual Health**. *J Am Plan Assoc* 2006, **72**.

176. Frank LD, Saelens BE, Powell KE, Chapman JE: **Stepping towards causation: do built environments or neighborhood and travel preferences explain physical activity, driving, and obesity?** *Social science & medicine (1982)* 2007, **65**.

177. Frank LD, Sallis JF, Conway TL, Chapman JE, Saelens BE, Bachman W: **Many pathways from land use to health - Associations between neighborhood walkability and active transportation, body mass index, and air quality**. *J Am Plan Assoc* 2007, **72**.

178. Glazier RH, Creatore MI, Weyman JT, Fazli G, Matheson FI, Gozdyra P, Moineddin R, Shriqui VK, Booth GL: **Density, Destinations or Both? A Comparison of Measures of Walkability in Relation to Transportation Behaviors, Obesity and Diabetes in Toronto, Canada**. *PloS one* 2014, **9**(1).

179. Hirsch JA, Diez Roux AV, Moore KA, Evenson KR, Rodriguez DA: **Change in walking and body mass index following residential relocation: the multi-ethnic study of atherosclerosis**. *American journal of public health* 2014, **104**(3):e49-56.

180. Hoehner CM, Handy SL, Yan Y, Blair SN, Berrigan D: **Association between neighborhood walkability, cardiorespiratory fitness and body-mass index**. *Social science & medicine* 2011, **73**(12):1707-1716.

181. Jilcott Pitts SB, McGuirt JT, Carr LJ, Wu Q, Keyserling TC: **Associations between body mass index, shopping behaviors, amenity density, and characteristics of the neighborhood food environment among female adult Supplemental Nutrition Assistance Program (SNAP) participants in eastern North Carolina**. *Ecology of food and nutrition* 2012, **51**(6):526-541.

182. King AC, Sallis JF, Frank LD, Saelens BE, Cain K, Conway TL, Chapman JE, Ahn DK, Kerr J: **Aging in neighborhoods differing in walkability and income: associations with physical activity and obesity in older adults**. *Social science & medicine (1982)* 2011, **73**(10):1525-1533.

183. Lim S, Harris TG: **Neighborhood Contributions to Racial and Ethnic Disparities in Obesity Among New York City Adults**. *American journal of public health* 2015, **105**(1):159-165.

184. Morinis J, Lebovic G, Gozdyra P, Khovratovich M, Carsley S, Creatore M, Moheddin R, Booth G, Maguire J, Parkin P *et al*: **The weight of place-the role of the neighbourhood in childhood obesity**. *Paediatrics and Child Health (Canada)* 2014, **19**(6):e40.

185. Muller-Riemenschneider F, Pereira G, Villanueva K, Christian H, Knuiman M, Giles-Corti B, Bull FC: **Neighborhood walkability and cardiometabolic risk factors in Australian adults: an observational study**. *BMC Public Health* 2013, **13**:755.

186. Oishi S, Saeki M, Axt J: **Are People Living in Walkable Areas Healthier and More Satisfied with Life?** *Applied psychology Health and well-being* 2015, **7**(3):365-386.

187. Sallis JF, Saelens BE, Frank LD, Conway TL, Slymen DJ, Cain KL, Chapman JE, Kerr J: **Neighborhood built environment and income: examining multiple health outcomes**. *Social science & medicine (1982)* 2009, **68**.

188. Smith KR, Brown BB, Yamada I, Kowaleski-Jones L, Zick CD, Fan JX: **Walkability and body mass index density, design, and new diversity measures**. *American journal of preventive medicine* 2008, **35**(3):237-244.

189. Smith KR, Zick CD, Kowaleski-Jones L, Brown BB, Fan JX, Yamada I: **Effects of neighborhood walkability on healthy weight: assessing selection and causal influences**. *Soc Sci Res* 2011, **40**.

190. Sriram U, LaCroix AZ, Barrington WE, Corbie-Smith G, Garcia L, Going SB, LaMonte MJ, Manson JE, Sealy-Jefferson S, Stefanick ML *et al*: **Neighborhood Walkability and Adiposity in the Women's Health Initiative Cohort**. *American journal of preventive medicine* 2016, **51**(5):722-730.

191. Van Cauwenberg J, Van Holle V, De Bourdeaudhuij I, Van Dyck D, Deforche B: **Neighborhood walkability and health outcomes among older adults: The mediating role of physical activity**. *Health & place* 2016, **37**:16-25.

192. Van Dyck D, Cerin E, Cardon G, Deforche B, Sallis JF, Owen N, de Bourdeaudhuij I: **Physical activity as a mediator of the associations between neighborhood walkability and adiposity in Belgian adults**. *Heal Place* 2010, **16**.

193. Wasfi RA, Dasgupta K, Orpana H, Ross NA: **Neighborhood Walkability and Body Mass Index Trajectories: Longitudinal Study of Canadians**. *American journal of public health* 2016, **106**(5):934-940.

194. Zick CD, Hanson H, Fan JX, Smith KR, Kowaleski-Jones L, Brown BB, Yamada I: **Re-visiting the relationship between neighbourhood environment and BMI: an instrumental variables approach to correcting for residential selection bias**. *Int J Behav Nutr Phys Act* 2013, **10**.

195. McDonald KN, Oakes JM, Forsyth A: **Effect of street connectivity and density on adult BMI: results from the Twin Cities Walking Study**. *Journal of epidemiology and community health* 2012, **66**.

196. Duncan GE, Cash SW, Horn EE, Turkheimer E: **Quasi-causal associations of physical activity and neighborhood walkability with body mass index: a twin study**. *Preventive medicine* 2015, **70**:90-95.

197. Gose M, Plachta-Danielzik S, Willie B, Johannsen M, Landsberg B, Muller MJ: **Longitudinal influences of neighbourhood built and social environment on children's weight status**. *International journal of environmental research and public health* 2013, **10**(10):5083-5096.

198. Michael YL, Gold R, Perrin N, Hillier TA: **Built environment and change in body mass index in older women**. *Health & place* 2013, **22**:7-10.

199. Sugiyama T, Niyonsenga T, Howard NJ, Coffee NT, Paquet C, Taylor AW, Daniel M: **Residential proximity to urban centres, local-area walkability and change in waist circumference among Australian adults**. *Preventive medicine* 2016, **93**:39-45.

200. Donatiello E, Dello Russo M, Formisano A, Lauria F, Nappo A, Reineke A, Sparano S, Barba G, Russo P, Siani A: **Physical activity, adiposity and urbanization level in children: Results for the italian cohort of the IDEFICS study**. *Public Health* 2013, **127**(8):761-765.

201. Burgoine T, Alvanides S, Lake AA: **Assessing the obesogenic environment of North East England**. *Heal Place* 2011, **17**.

202. Nayha S, Lankila T, Rautio A, Koiranen M, Tammelin TH, Taanila A, Rusanen J, Laitinen J: **Body mass index and overweight in relation to residence distance and population density: experience from the Northern Finland birth cohort 1966**. *BMC Public Health* 2013, **13**:938.

203. Penney TL, Rainham DG, Dummer TJ, Kirk SF: **A spatial analysis of community level overweight and obesity**. *Journal of human nutrition and dietetics : the official journal of the British Dietetic Association* 2014, **27 Suppl 2**:65-74.

204. Poortinga W: **Perceptions of the environment, physical activity, and obesity**. *Social science & medicine (1982)* 2006, **63**.

205. Lahti-Koski M, Taskinen O, Simila M, Mannisto S, Laatikainen T, Knekt P, Valsta LM: **Mapping geographical variation in obesity in Finland**. *European journal of public health* 2008, **18**(6):637-643.

206. Moore JB, Brinkley J, Crawford TW, Evenson KR, Brownson RC: **Association of the built environment with physical activity and adiposity in rural and urban youth**. *Preventive medicine* 2013, **56**(2):145-148.

207. Boehmer TK, Hoehner CM, Deshpande AD, Brennan Ramirez LK, Brownson RC: **Perceived and observed neighborhood indicators of obesity among urban adults**. *Int J Obes* 2007, **31**.

208. Knapp EA, Nau C, Brandau S, DeWalle J, Hirsch AG, Bailey-Davis L, Schwartz BS, Glass TA: **Community Audit of Social, Civil, and Activity Domains in Diverse Environments (CASCADDE)**. *American journal of preventive medicine* 2017.

209. Powell-Wiley TM, Ayers CR, de Lemos JA, Lakoski SG, Vega GL, Grundy S, Das SR, Banks-Richard K, Albert MA: **Relationship between perceptions about neighborhood environment and prevalent obesity: data from the Dallas Heart Study**. *Obesity (Silver Spring, Md)* 2013, **21**(1):E14-21.

210. Rundle A, Diez Roux AV, Free LM, Miller D, Neckerman KM, Weiss CC: **The urban built environment and obesity in New York City: a multilevel analysis**. *Am J Heal Promot* 2007, **21**.

211. Duncan DT, Castro MC, Gortmaker SL, Aldstadt J, Melly SJ, Bennett GG: **Racial differences in the built environment--body mass index relationship? A geospatial analysis of adolescents in urban neighborhoods**. *International journal of health geographics* 2012, **11**:11.

212. Arcaya M, James P, Rhodes JE, Waters MC, Subramanian SV: **Urban sprawl and body mass index among displaced Hurricane Katrina survivors**. *Preventive medicine* 2014, **65**:40-46.

213. Ewing R, Meakins G, Hamidi S, Nelson AC: **Relationship between urban sprawl and physical activity, obesity, and morbidity - update and refinement**. *Health & place* 2014, **26**:118-126.

214. Ewing R, Schmid T, Killingsworth R, Zlot A, Raudenbush S: **Relationship between urban sprawl and physical activity, obesity, and morbidity**. *Am J Heal Promot* 2003, **18**.

215. James P, Troped PJ, Hart JE, Joshu CE, Colditz GA, Brownson RC, Ewing R, Laden F: **Urban Sprawl, Physical Activity, and Body Mass Index: Nurses’ Health Study and Nurses' Health Study II**. *Am J Public Heal* 2012, **103**.

216. Joshu CE, Boehmer TK, Brownson RC, Ewing R: **Personal, neighbourhood and urban factors associated with obesity in the United States**. *J Epidemiol Community Heal* 2008, **62**.

217. Plantinga AJ, Bernell S: **The association between urban sprawl and obesity: Is it a two-way street?** *J Reg Sci* 2007, **47**.

218. Eid J, Overman HG, Puga D, Turner MA: **Fat city: Questioning the relationship between urban sprawl and obesity**. *J Urban Econ* 2008, **63**.

219. King TL, Bentley RJ, Thornton LE, Kavanagh AM: **Using kernel density estimation to understand the influence of neighbourhood destinations on BMI**. *BMJ open* 2016, **6**(2).

220. Oliver M, Witten K, Blakely T, Parker K, Badland H, Schofield G, Ivory V, Pearce J, Mavoa S, Hinckson E *et al*: **Neighbourhood built environment associations with body size in adults: mediating effects of activity and sedentariness in a cross-sectional study of New Zealand adults**. *BMC Public Health* 2015, **15**:956.

221. Norman GJ, Adams MA, Kerr J, Ryan S, Frank LD, Roesch SC: **A latent profile analysis of neighborhood recreation environments in relation to adolescent physical activity, sedentary time, and obesity**. *Journal of public health management and practice : JPHMP* 2010, **16**(5):411-419.

222. Ross NA, Tremblay S, Khan S, Crouse D, Tremblay M, Berthelot J-M: **Body mass index in urban Canada: neighborhood and metropolitan area effects**. *American journal of public health* 2007, **97**(3):500-508.

223. Cunningham-Myrie CA, Theall KP, Younger NO, Mabile EA, Tulloch-Reid MK, Francis DK, McFarlane SR, Gordon-Strachan GM, Wilks RJ: **Associations between neighborhood effects and physical activity, obesity, and diabetes: The Jamaica Health and Lifestyle Survey 2008**. *Journal of clinical epidemiology* 2015, **68**(9):970-978.

224. Leonardi C, Simonsen NR, Yu Q, Park C, Scribner RA: **Street Connectivity and Obesity Risk: Evidence From Electronic Health Records**. *American journal of preventive medicine* 2017, **52**(1s1):S40-s47.

225. Samimi A, Mohammadian A, Madanizadeh S: **Effects of transportation and built environment on general health and obesity**. *Transp Res Part D-Transport Environ* 2009, **14**.

226. Brown BB, Yamada I, Smith KR, Zick CD, Kowaleski-Jones L, Fan JX: **Mixed land use and walkability: Variations in land use measures and relationships with BMI, overweight, and obesity**. *Health & place* 2009, **15**(4):1130-1141.

227. James P, Berrigan D, Hart JE, Hipp JA, Hoehner CM, Kerr J, Major JM, Oka M, Laden F: **Effects of buffer size and shape on associations between the built environment and energy balance**. *Health & place* 2014, **27**:162-170.

228. De Bourdeaudhuij I, Van Dyck D, Salvo D, Davey R, Reis RS, Schofield G, Sarmiento OL, Mitas J, Christiansen LB, MacFarlane D *et al*: **International study of perceived neighbourhood environmental attributes and Body Mass Index: IPEN Adult study in 12 countries**. *Int J Behav Nutr Phys Act* 2015, **12**:62.

229. Frank LD, Andresen MA, Schmid TL: **Obesity relationships with community design, physical activity, and time spent in cars**. *American journal of preventive medicine* 2004, **27**.

230. Pouliou T, Elliott SJ: **Individual and socio-environmental determinants of overweight and obesity in Urban Canada**. *Health & place* 2010, **16**(2):389-398.

231. Pouliou T, Elliott SJ, Paez A, Newbold KB: **Building obesity in Canada: understanding the individual- and neighbourhood-level determinants using a multi-level approach**. *Geospatial health* 2014, **9**(1):45-55.

232. Rutt CD, Coleman KJ: **Examining the relationships among built environment, physical activity, and body mass index in El Paso, TX**. *Preventive medicine* 2005, **40**(6):831-841.

233. Littenberg B, Bonnell LN, LeBruin AS, Lubetkin DA, Troy AR, Zia A: **The Relationship Between Access to Natural Environmental Amenities and Obesity**. *Cureus* 2015, **7**(11):e377.

234. Bodea TD, Garrow LA, Meyer MD, Ross CL: **Socio-demographic and built environment influences on the odds of being overweight or obese: The Atlanta experience**. *Transp Res Part a-Policy Pract* 2009, **43**.

235. King AC, Salvo D, Banda JA, Ahn DK, Gill TM, Miller M, Newman AB, Fielding RA, Siordia C, Moore S *et al*: **An observational study identifying obese subgroups among older adults at increased risk of mobility disability: do perceptions of the neighborhood environment matter?** *Int J Behav Nutr Phys Act* 2015, **12**:157.

236. Pendola R, Gen S: **BMI, auto use, and the urban environment in San Francisco**. *Health & place* 2007, **13**.

237. Zhao Z, Kaestner R: **Effects of urban sprawl on obesity**. *J Heal Econ* 2010, **29**.

238. Brown BB, Werner CM, Smith KR, Tribby CP, Miller HJ: **Physical activity mediates the relationship between perceived crime safety and obesity**. *Preventive medicine* 2014, **66**:140-144.

239. Christian H, Giles-Corti B, Knuiman M, Timperio A, Foster S: **The influence of the built environment, social environment and health behaviors on body mass index. results from RESIDE**. *Preventive medicine* 2011, **53**(1-2):57-60.

240. Nogueira H, Ferrao M, Gama A, Mourao I, Marques VR, Padez C: **Perceptions of neighborhood environments and childhood obesity: Evidence of harmful gender inequities among Portuguese children**. *Health & place* 2013, **19**:69-73.

241. Sanchez-Cruz JJ, de Ruiter I, Jimenez-Moleon JJ: **Individual, family and environmental factors associated with pediatric excess weight in Spain: a cross-sectional study**. *Bmc Pediatrics* 2014, **14**.

242. Li K, Wen M, Henry KA: **Residential racial composition and black-white obesity risks: differential effects of neighborhood social and built environment**. *International journal of environmental research and public health* 2014, **11**(1):626-642.

243. Ball K, Lamb K, Travaglini N, Ellaway A: **Street connectivity and obesity in Glasgow, Scotland: impact of age, sex and socioeconomic position**. *Health & place* 2012, **18**(6):1307-1313.

244. Wang F, Wen M, Xu Y: **Population-Adjusted Street Connectivity, Urbanicity and Risk of Obesity in the U.S**. *Applied geography (Sevenoaks, England)* 2013, **41**:1-14.

245. Li W, Dorans KS, Wilker EH, Rice MB, Schwartz J, Coull BA, Koutrakis P, Gold DR, Fox CS, Mittleman MA: **Residential proximity to major roadways, fine particulate matter, and adiposity: The framingham heart study**. *Obesity (Silver Spring, Md)* 2016, **24**(12):2593-2599.

246. Van Hulst A, Gauvin L, Kestens Y, Barnett TA: **Neighborhood built and social environment characteristics: a multilevel analysis of associations with obesity among children and their parents**. *International journal of obesity (2005)* 2013, **37**(10):1328-1335.

247. Garden FL, Jalaludin BB: **Impact of urban sprawl on overweight, obesity, and physical activity in Sydney, Australia**. *J Urban Heal* 2008, **86**.

248. Lopez R: **Urban sprawl and risk for being overweight or obese**. *American journal of public health* 2004, **94**(9):1574-1579.

249. O'Dare Wilson K: **Place matters: Mitigating obesity with the person-in-environment approach**. *Social work in health care* 2016, **55**(3):214-230.

250. McTigue KM, Cohen ED, Moore CG, Hipwell AE, Loeber R, Kuller LH: **Urban Neighborhood Features and Longitudinal Weight Development in Girls**. *American journal of preventive medicine* 2015, **49**(6):902-911.

251. Morris DS, Main EC, Harris JK, Moland A, Cude C: **State-Issued Identification Cards Reveal Patterns in Adult Weight Status**. *International journal of environmental research and public health* 2015, **12**(6):6388-6402.

252. Oka M, Link CL, Kawachi I: **Area-based variations in obesity are more than a function of the food and physical activity environment : area-based variations in obesity**. *Journal of urban health : bulletin of the New York Academy of Medicine* 2013, **90**(3):442-463.

253. Rundle A, Field S, Park Y, Freeman L, Weiss CC, Neckerman K: **Personal and neighborhood socioeconomic status and indices of neighborhood walk-ability predict body mass index in New York City**. *Social science & medicine (1982)* 2008, **67**.

254. Alfonzo M, Guo Z, Lin L, Day K: **Walking, obesity and urban design in Chinese neighborhoods**. *Preventive medicine* 2014, **69 Suppl 1**:S79-85.

255. Leonard T, Powell-Wiley TM, Ayers C, Murdoch JC, Yin W, Pruitt SL: **Property Values as a Measure of Neighborhoods: An Application of Hedonic Price Theory**. *Epidemiology (Cambridge, Mass)* 2016, **27**(4):518-524.

256. Gaglioti AH, Petterson S, Bazemore A, Phillips R: **Access to Primary Care in US Counties Is Associated with Lower Obesity Rates**. *Journal of the American Board of Family Medicine : JABFM* 2016, **29**(2):182-190.
