# Supplementary material for: Using data from online geocoding services for the assessment of environmental obesogenic factors: a feasibility study
Source: Int J Health Geogr. 2019 Jun 7;18:13. doi: 10.1186/s12942-019-0177-9 (PMC6555943; doi:10.1186/s12942-019-0177-9)
Supplement: Supplementary file 2 — Additional file 2: Table S2. Complete list of chosen OSM variables. [file 12942_2019_177_MOESM2_ESM.doc]

Additional file 2: Complete list of chosen OSM variables

Table S2: Complete list of chosen OSM variables (Key = Value)

"amenity"="bar"

"amenity"="bbq"

"amenity"="biergarten"

"amenity"="cafe"

"amenity"="fast_food"

"amenity"="food_court"

"amenity"="ice_cream"

"amenity"="pub"

"amenity"="restaurant"

"amenity"="college"

"amenity"="school"

"amenity"="bicycle_parking"

"amenity"="bicycle_rental"

"amenity"="boat_sharing"

"amenity"="bus_station"

"amenity"="motorcycle_parking"

"amenity"="parking"

"amenity"="taxi"

"amenity"="clinic"

"amenity"="dentist"

"amenity"="doctors"

"amenity"="hospital"

"amenity"="nursing_home"

"amenity"="pharmacy"

"amenity"="dive_centre"

"amenity"="dojo"

"amenity"="hunting_stand"

"amenity"="kneipp_water_cure"

"amenity"="marketplace"

"amenity"="ranger_station"

"amenity"="vending_machine"

"leisure"="beach_resort"

"leisure"="dance"

"leisure"="dog_park"

"leisure"="fishing"

"leisure"="fitness_centre"

"leisure"="garden"

"leisure"="golf_course"

"leisure"="ice_rink"

"leisure"="nature_reserve"

"leisure"="park"

"leisure"="pitch"

"leisure"="playground"

"leisure"="sports_centre"

"leisure"="stadium"

"leisure"="swimming_area"

"leisure"="swimming_pool"

"leisure"="track"

"leisure"="water_park"

"sport"

"landuse"="allotments"

"landuse"="farmland"

"landuse"="farmyard"

"landuse"="forest"

"landuse"="grass"

"landuse"="greenfield"

"landuse"="greenhouse_horticulture"

"landuse"="meadow"

"landuse"="orchard"

"landuse"="plant_nursery"

"landuse"="recreation_ground"

"landuse"="village_green"

"landuse"="vineyard"

"natural"="wood"

"natural"="tree_row"

"natural"="tree"

"natural"="scrub"

"natural"="grassland"

"natural"="fell"

"natural"="water"

"office"="therapist"

"shop"="bakery"

"shop"="beverages"

"shop"="butcher"

"shop"="cheese"

"shop"="chocolate"

"shop"="coffee"

"shop"="confectionery"

"shop"="convenience"

"shop"="deli"

"shop"="dairy"

"shop"="farm"

"shop"="greengrocer"

"shop"="ice_cream"

"shop"="pasta"

"shop"="pastry"

"shop"="seafood"

"shop"="spices"

"shop"="tea"

"shop"="wine"

"shop"="supermarket"

"shop"="medical_supply"

"shop"="nutrition_supplements"

"shop"="garden_centre"

"shop"="garden_furniture"

"shop"="bicycle"

"shop"="fishing"

"shop"="free_flying"

"shop"="hunting"

"shop"="outdoor"

"shop"="scuba_diving"

"shop"="sports"

"shop"="swimming_pool"

"vending"="bicycle_tube"

"vending"="bread"

"vending"="chemist"

"vending"="chewing_gums"

"vending"="coffee"

"vending"="drinks"

"vending"="first_aid"

"vending"="fishing_tackle"

"vending"="food"

"vending"="ice_cream"

"vending"="milk"

"vending"="sweets"

"craft"="bakery"

"craft"="caterer"

"tourism"="aquarium"

"tourism"="camp_site"

"tourism"="picnic_site"

"tourism"="theme_park"

"highway"="bus_stop"

"highway"="rest_area"

"railway"="station"

"aeroway"="heliport"

"aeroway"="aerodrome"

Note: Key mention without corresponding value means that all values listed within this category were chosen
